# Synthesis, molecular docking and biological evaluation of 2,4-diaminopyrimidines and pyrazoles as aurora kinase inhibitors

**DOI:** 10.17179/excli2026-9347

**Published:** 2026-08-09

**Authors:** Kaksha Sankhe, Mrunal Jadhav, Arati Prabhu, Tabassum Khan

**Affiliations:** 1 Department of Pharmaceutical Chemistry, SVKM's Dr. Bhanuben Nanavati College of Pharmacy, University of Mumbai, Mumbai, Maharashtra, India

**Keywords:** Aurora kinase, cancer, pyrazoles, pyrimidines, Aurora kinase inhibitor

## Abstract

Aurora kinases (*AURKs*) constitute a family of serine/threonine protein kinases that regulate cell cycle progression and are frequently overexpressed in various cancers, thereby contributing to tumorigenesis. A series of pyrazoles and 2,4-diaminopyrimidines were designed and synthesized as potential cytotoxic entities in ovarian, brain, and leukemia cancer. Molecular docking studies were conducted on *AURKA* (PDB IDs: 3UP7) and *AURKB *(PDB IDs: 4AF3). Compounds exhibiting favorable interaction with the ATP binding site and key residues (Ala213 and Glu211 in *AURKA;* Ala157 and Glu155 in *AURKB*) were identified as potential candidates. Compounds 2j, 2l, 2p, 9a, and 10a showed dose-dependent cytotoxicity in SKOV3, C6, and THP1 cell lines. Of them, 2l, 9a, and 10a effectively suppressed the growth of SKOV3, C6, and THP1 cells with IC_50 _values of 8.63 µM, 0.019 µM and 0.084 µM, respectively. Compounds 2l, 2p, 9a, and 10a showed promising inhibitory activity in *AURKA* and *AURKB*. 2p and 10a exhibited comparable potency to the standard Barasertib at 10 µM (2p: 68.14 %, 10a: 55.64 %, Barasertib: 78.42 %) highlighting their potential as novel therapeutic agents. The compounds 2j and 2p displayed potent inhibitory activity in *AURKB. *At 10µM, these compounds achieved inhibition rates comparable to standard Barasertib (2j: 56.36 %, 2p: 56.39 %, Barasertib: 68.62 %). Molecular docking and ADME prediction studies indicated these compounds to have good drug likeliness scores. The results of this study indicated that 2l, 2p, 9a, and 10a can be used as lead compounds targeting *AURK* for development as anti-cancer agents.

See also the graphical abstract(Fig. 1).

## Introduction

**Figure 2:**
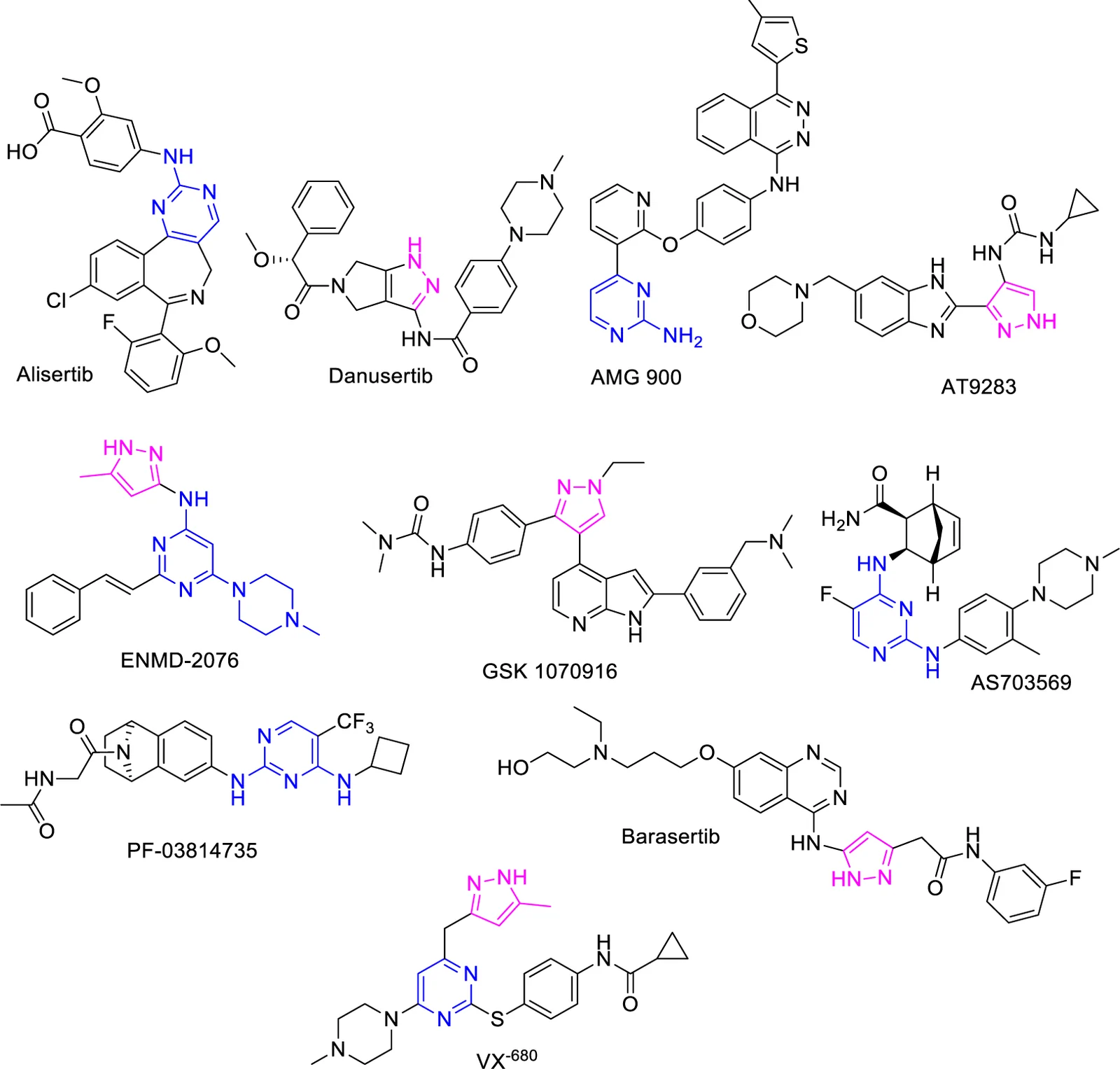
*AURK* inhibitors in clinical trials

Cancer is the world's leading cause of death. In 2022, almost 20 million people were affected by cancer and nearly 10 million died from it. This is expected to grow by about 77 % by 2050 as per World Health Organisation (WHO), putting a big strain on healthcare systems and communities. Cancer is uncontrolled growth and spread of abnormal cells and can lead to invasion of nearby tissues and metastasis to distant organs. The limitations of current cancer therapies, including side effects and emerging drug resistance, which highlight the need for innovative and effective therapies via drug discovery and drug delivery system that can impede cancer progression (WHO, 2023[18]). Aurora kinases (*AURK*s) are a family of three highly similar serine/threonine protein kinases, and include Aurora kinase A (*AURKA*), Aurora kinase B (*AURKB*), and Aurora kinase C (*AURK*C) (Bolanos-Garcia, 2005[3]). These kinases play critical role in various stages of mitosis, such as centrosome maturation, chromosome segregation, and cytokinesis. Dysregulation of *AURK* can lead to aneuploidy and cell death. *AURKA* is involved in centrosome duplication and spindle assembly. It is found on chromosome 20q13.2 (a region amplified in various cancers and expressed in early mitosis at G2/M phase) and interacts with proteins like TPX2 (targeting protein for Xenopus kinesin-like protein-2) and Ajuba to initiate its activation. Overexpression of *AURKA* is linked to colon, ovarian, breast, and pancreatic cancer (Du et al., 2021[5]). *AURKB* is located on 17p13 chromosomes and acts as a part of the chromosomal passenger complex (CPC) with survivin, borealin, and inner centromere protein (INCENP). The CPC is crucial for accurate chromosome alignment and segregation during mitosis. Overexpression of *AURKB* is associated with glioblastoma, oral cancer, lung cancer, and prostate cancer (Kovacs et al., 2023[7]). *AURK*C is primarily expressed in the testes, and is involved in spermatogenesis. It is located on 19q13.43 chromosomes and plays a role in meiosis (Quartuccio and Schindler, 2015[8]). Over the past decade, *AURK* inhibitors have emerged as promising anti-cancer agents. The overexpression of these kinases in various cancer types has fueled the development of targeted therapies. Numerous *AURK* inhibitors such as AMG900, AZD1152 (Barasertib), AT9283, MLN8237 (Alisertib), MLN8054, MK-0457 (VX-680), PF-03814735, ENMD-2076, PHA-739358 (Danusertib), GSK1070916, and AS703569 (Figure 2(Fig. 2)) are currently in various phases of clinical trials, demonstrating their potential to effectively treat a range of cancers. Heterocyclic compounds have emerged as privileged scaffolds in the realm of anticancer drug discovery. Their ability to interact with diverse biological targets and modulate key metabolic pathways and cellular processes within cancer cells renders them highly appealing candidates for development of new therapeutic strategies. Recent research has identified various scaffolds such as 2-aminopyrimidine, 2,4-diaminopyrimidine, benzimidazole, indazole, quinoline, pyrrolopyrimidin, quinoline, pyrazole, and phthalazinone to effectively inhibit *AURK*lchs. These compounds often mimic adenosine and bind competitively to the ATP-binding site, suggesting a promising mechanism of action for disrupting *AURK* function. Clinical studies have revealed that 2-aminopyrimidin, 2,4-diaminopyrimidine, and pyrazole scaffolds comprise approximately 50 % of the novel molecular entities under investigation. The majority of *AURKA* and *AURKB* inhibitors under research possess dual ability to block and mimic the ATP binding site (Sankhe et al., 2021[12]). By obstructing ATP binding to the active site, these inhibitors can effectively reduce kinase activity, leading to cell death and potentially halting the proliferation of aggressive cancer cells. The aim of this project was to develop a series of 2, 4-diaminopyrimidine and pyrazole-based compounds targeting *AURKA* and *AURKB* since their structural attribute, promote interactions at the active site important amino acids residues at the hinge region of the enzyme. These compounds were synthesized and evaluated for *in-vitro *cytotoxic activity in ovarian, glioblastoma, and leukemia cell lines. 

## Results and Discussion

### Chemistry

A series of 2,4-diaminopyrimidines **(2a-2p)** were synthesized via a nucleophilic substitution reaction as outlined in Scheme 1 (all schemes will follow later in the text). The reaction involved displacement of the chlorine in 2-amino-4-chloro-5-methyl-pyrimidines 1 with various amines (Scheme 2). The yield (60 % to 75 %) and time (24-30 hrs) varied depending on the amine substituent. The completion of the reaction was monitored using thin layer chromatography (TLC) comprising of *n-*Hexane: ethyl acetate (3:7) as mobile phase. The molar ratio of the substituted amines varied with respect to 2-amino-4-chloro-5-methyl-pyrimidines 1 among all synthesized compounds (see later, Table 6). Cyclic amines, such as pyrrolidine, piperidine, morpholine, methyl piperazine, and ethyl piperazine, were readily substituted due to their lack of double bonds and the increased availability of the nitrogen lone pair. The higher basicity of these amines further enhanced their nucleophilic reactivity. Aromatic amines, like methyl aniline, generally exhibited more favorable reaction profiles compared to fluoro aniline. The electron-donating methyl group in methyl aniline increased the basicity of the amine, making it a stronger nucleophile. Conversely, the electron-withdrawing fluorine atom in fluoro aniline reduced its nucleophilicity, leading to longer reaction times. These findings demonstrate the influence of amine structure on the nucleophilic substitution reaction. The steric hindrance and electronic properties of the amine substituents significantly impacted the reaction efficiency. Understanding these factors is crucial for optimizing the synthesis of 2,4-diaminopyrimidines and related compounds. The synthesized compounds were subjected to ultra-fast liquid chromatography (UFLC) analysis to assess their purity. The result indicated a purity range of 80-100 %, confirming their suitability for subsequent spectroscopic characterization and biological evaluation. 

Fourier-transform infrared (FT-IR) spectroscopy was employed to identify key functional groups. Characteristics peaks were observed for 2,4-diaminopyrimidine series **(2a-2p)** approximately at 3300-3350 cm^-1 ^for NH_2 _stretching, 2914-2988 cm^-1^ for -CH stretching, and 1200-1350 cm^-1^ for C=N stretching, confirming the presence of these functional groups. Proton nuclear magnetic resonance (*^1^*H NMR) spectroscopy provided valuable insights into structural features of the synthesized compounds **(2a-2p)**. The pyrimidine proton at the 5^th^ carbon position in 2,4-diaminopyrimidines series resonated as a characteristic singlet at 5.82-5.96 ppm. The amine protons typically appeared as singlets in the range of 4.5-6.0 ppm. When the amine substituent contained aliphatic groups, signals were observed in the aliphatic region (1-3 ppm). This upfield shift is due to the shielding effect of the aliphatic groups as the electron cloud around the aliphatic group reduces effective magnetic field experienced by protons. Aromatic protons associated with phenyl groups were detected as a multiplets in the range of 6.5-7.5 ppm. Carbon 13 nuclear magnetic resonance (C^13^ NMR) spectroscopy of **(2a-2p)** was utilized to examine the carbon framework of the molecules. The aromatic carbons of the pyrimidine ring typically resonated in the downfield region (160-170 ppm) due to their electron-deficient nature. Aliphatic carbons in the amine substituent appeared in the range of 15-20 ppm, while phenyl aromatic carbons resonated between 120-130 ppm. 

2-amino-4-hydroxypyrimidine **5** was synthesized via a condensation reaction between guanidine hydrochloride and ethyl acetoacetate, achieving a yield of 70-75 % with 300^ °^C melting point (Scheme 3 and Scheme 4). The reaction progress was monitored using TLC with chloroform: Methanol (9.6:0.4) as the mobile phase. The FTIR of compound** 5** confirmed the presence of key functional groups, including -NH_2 _stretching at 3321 cm-1, stretching at 3062 cmh^-1^, 1649 cm^-1^ for C=C stretch, and 2927 cm^-1^ for -CH stretching. UFLC analysis indicated 100 % purity of the compound, and mass spectroscopy (MS) determined molecular weight to be 126 m/z. The ^1^H NMR spectroscopy revealed characteristics signals of compound **5 **for the amino group (singlet at 6.727 ppm), pyrimidine proton (singlet at 5.355 ppm), and methyl group (singlet at 2.018 ppm). The C^13^ NMR spectroscopy of compound** 5** confirmed the presence of the amino group (resonance at 146.96 ppm), hydroxyl group (resonance at 165.92 ppm), and pyrimidine ring carbons (resonance in the downfield region, 150-170 ppm). The methyl group carbon appeared in the upfield region at 23.89 ppm due to its shielding effect. Following characterization, compound **5** was subjected to *in-vitro* biological evaluation.

Acetophenone phenylhydrazones **8** were synthesized in 70-80 % yield by reacting acetophenones with phenyl hydrazine (Scheme 5). The reaction progress was monitored using TLC with hexane: ethyl acetate (9:1) as the mobile phase, and melting point was determined to be 106^o^C. Subsequently, vilsmeier haack formylation using phosphorous oxychloride (POCL_3_) and N, N- dimethylformamide DMF afforded pyrazole-4-carbaldehydes **(9a-9e)** in 60-70 % yield (Scheme 6 and Scheme 7). The TLC analysis with *n-*hexane: ethyl acetate (9:1) and melting point determination (136-140^o^C) confirmed completion of reaction. MS spectroscopy was employed to determine the exact molecular weights of the synthesized compounds. The FTIR spectrum revealed characteristic peaks of compound **9a-9e** at the 3124 cm^-1^ for -CH stretching, 1523 cm^-1^ for C=N stretching, and 1655 cm^-1^ for C=O stretching. ^1^H NMR of pyrazole-4-carbaldehydes **(9a-9e)** exhibited characteristic signals for the aldehyde proton (singlet at 9.73-10.05 ppm) due to its deshielding effect. The proton on pyrazole ring appears as a singlet in the region of 8.40-8.53 ppm and aromatic protons (multiplets at 7.45-7.82 ppm) in case of phenyl substituted pyrazoles. ^13^C NMR spectroscopy of compounds **9a-9e** confirmed the presence of the aldehyde proton (resonance at 185-190 ppm), pyrazole ring carbons (resonance at 130-150 ppm) and aromatic carbons (resonance at 120-130 ppm) in phenyl substituted pyrazole. 

The compounds pyrazole-4-carboxylic acids **(10a-10c)** were synthesized by oxidizing pyrazole-4-carbaldehyde with potassium permanganate (KMnO_4_) (Scheme 8 and Scheme 9). The reaction yielded 60-80 % of desired products, transforming the dark purple solution to the dark brown solution. UFLC analysis confirmed the high purity (90-100 %) of the synthesized compound prior to biological evaluation. The reaction progress was monitored using TLC with hexane: ethyl acetate (6:4) as the mobile phase and melting point was determined to be 170-180^o^C. MS **(10a-10c)** was employed to determine the exact molecular weights. FTIR spectroscopy of compound **10a-10c** revealed characteristics broad stretch at 3428 cm^-1^ for hydroxyl group and 1677 cm^-1^ for the carbonyl group. ^1^H NMR of pyrazole-4-carboxylic acids **(10a-10c)** exhibited characteristic signals for the pyrazole ring proton (singlet at 8.40-8.53 ppm) and aromatic protons (multiplets at 7.45-7.90 ppm) in the case of phenyl substituted pyrazoles. ^13^C NMR spectroscopy of compound** 10a-10c** confirmed the presence of the carboxylic acid carbonyl carbon (resonance at 169-171 ppm), pyrazole ring carbons (resonance at 138-140 ppm), and aromatic carbons (resonance at 120-130 ppm) in phenyl substituted pyrazoles.

### Biological evaluation

#### In-vitro cytotoxic activity

The cytotoxic activity of synthesized compounds was evaluated in *AURK* overexpressing cell lines like ovarian cancer (SK), brain cancer (C6), and Leukemia (THP1) using the 3-(4,5-dimethylthiazol-2-yl)-2,5-diphenyltetrazolium bromide (MTT) assay. Compounds were tested at a concentration range of 0.1 to1000 µM. Gemcitabine 50µM, doxorubicin (0.1-1000nM), and paclitaxel (50nM) served as reference standards for SKOV3, C6, and THP1, respectively. Our findings suggested that several synthesized compounds induced significant concentration dependant cytotoxicity across all cell lines (Table 1(Tab. 1)). Notably, 2, 4-diaminopyrimidines derivatives exhibited potent activity in THP1 cells, followed by C6 and SKOV3 cells. Conversely, the pyrazole derivatives have shown activity against THP1, followed by the C6 cell line. Specifically, compounds 2l and 2m showed promising activity in SKOV3 cells with IC_50_ 8.63±0.05 and 62.57±13.220 µM respectively. For C6 cells, compounds 2k, 2l, 9a and 9b compounds exhibited promising cytotoxicity with IC_50 _12.75±0.012, 0.49±0.001, 0.019±0.015, and 0.619±0.012 µM, respectively. Similarly, 2j, 2l, 2p, 9a, and 10a showed potent cytotoxic activity in THP1 cells with IC_50_ 16.59±0.039, 0.18±0.026, 1.26±18.201, 0.64±56.358, and 0.084±0.030 µM respectively. To validate the target specificity of these compounds, we performed *AURK *target inhibition studies. The most active compounds, based on their IC_50 _were tested in *AURKA *and *AURKB *enzyme. 

#### In-vitro AURKA and AURKB kinase inhibition assay

**Figure 3:**
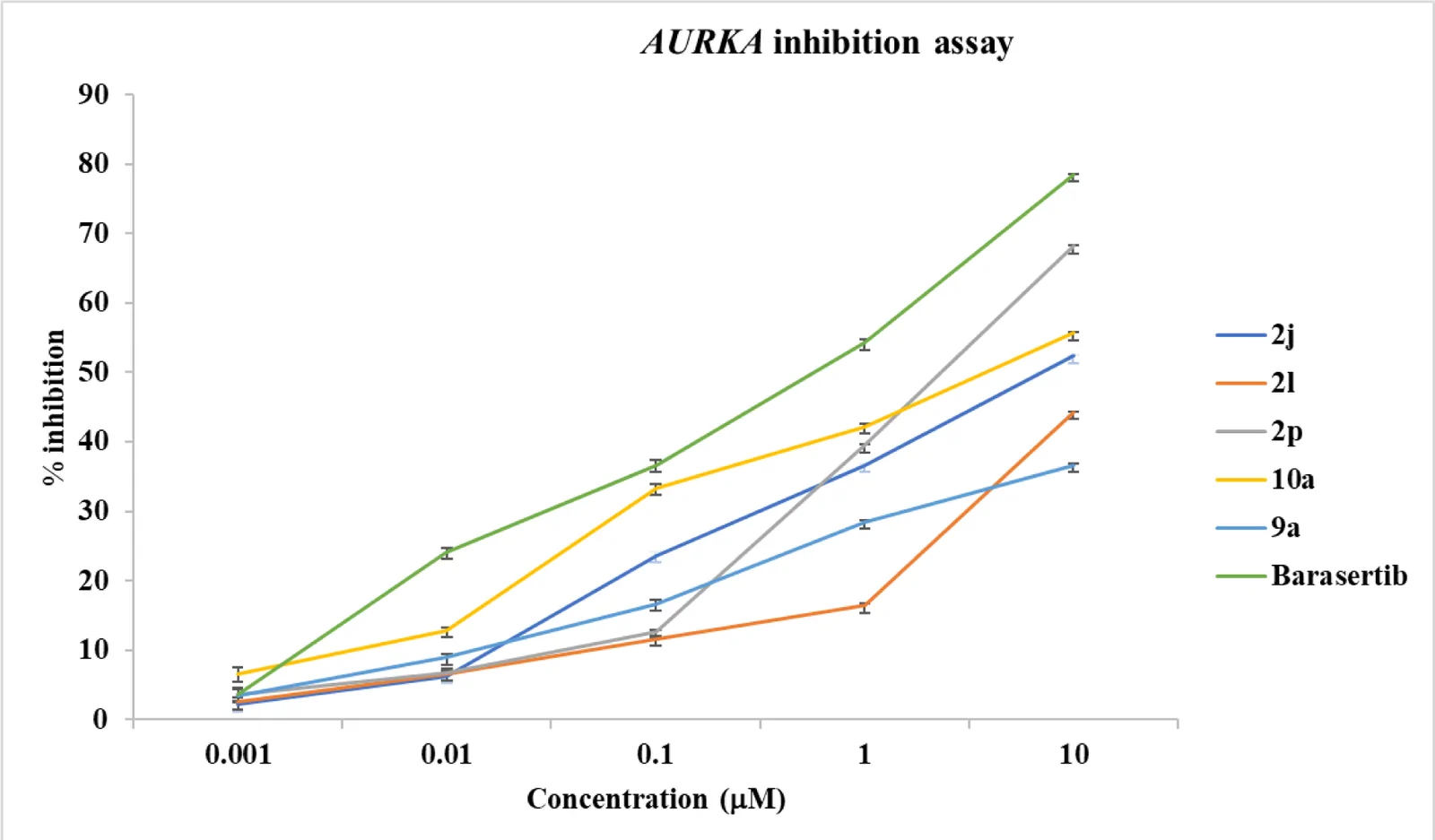
*AURKA* inhibition assay

**Figure 4:**
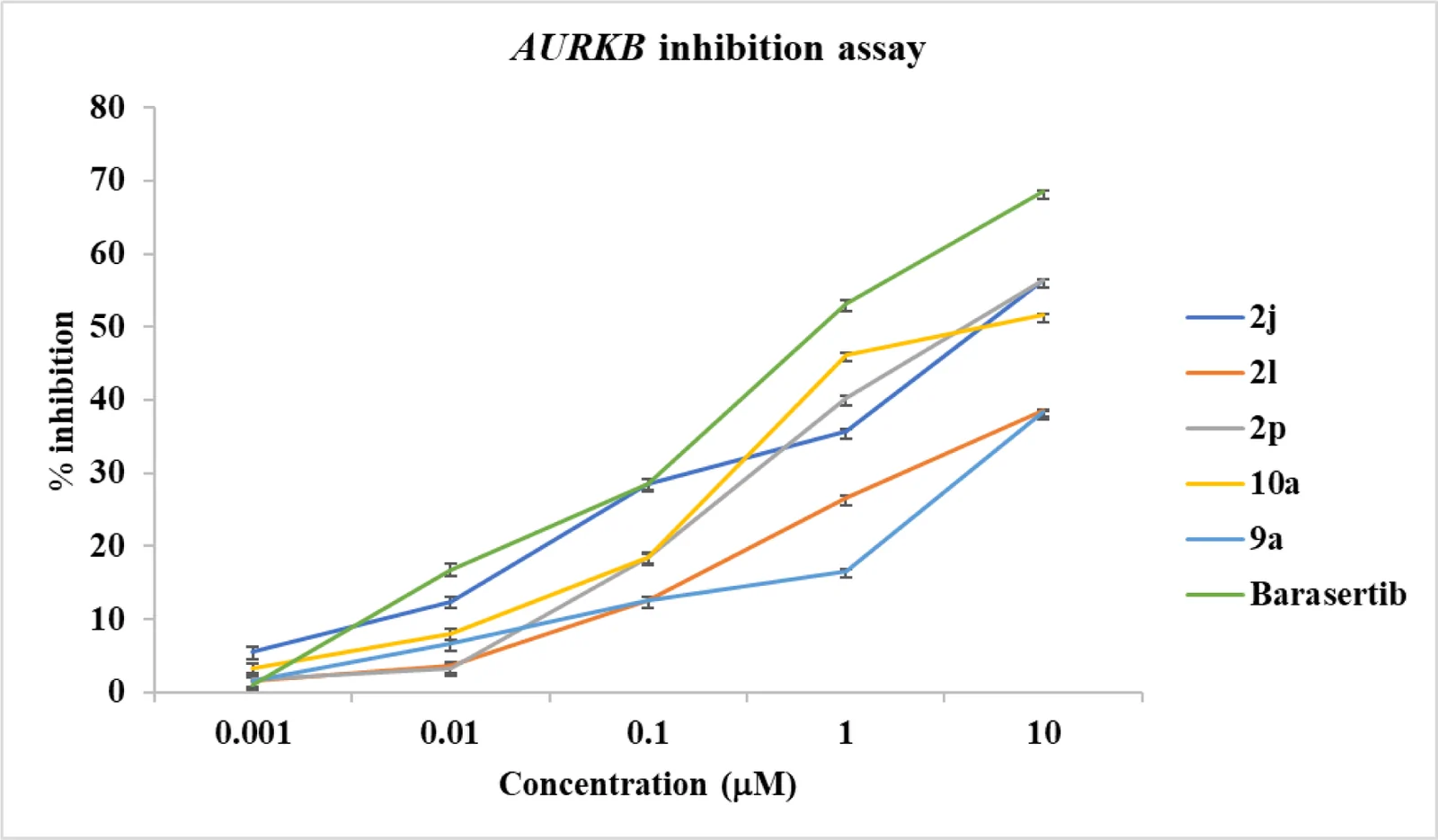
*AURKB* inhibition assay

*AURK* inhibition assay was performed to elucidate the molecular mechanism underlying the observed cytotoxicity. Compounds 2j, 2l, 2p, 9a, and 10a exhibited potent cytotoxicity were selected for further investigation. In silico molecular docking studies suggested that these compounds could bind to the hinge region of the kinase domain, forming hydrogen bonds, aromatic hydrogen bonds, and π-π interactions with key amino acids residues. Barasertib, a phase III drug, served as a reference standard in this study. Compounds 2p, 10a, and 2j exhibited significant inhibition of both *AURKA* and *AURKB*. At concentration 10µM, these compounds demonstrated inhibition percentage of 68.14 %, 55.64 %, and 52.36 % in *AURKA,* respectively, compared to 78.42 % for the standard inhibitor, Barasertib (Figure 3(Fig. 3)). Similarly, in *AURKB* 2j, 2p, and 10a showed inhibition percentage of 56.36 %, 56.39 %, and 51.63 %, respectively, compared to 68.62 1 % for Barasertib (Figure 4(Fig. 4)). Further analysis of IC_50_ (Table 2(Tab. 2)) revealed that compounds 2p and 10a exhibited greater potency in *AURKA*, with IC50 of 1 µM, 2 µM, respectively, compared to Barasertib 0.6 µM. In contrast, 2j, 2p, and 10a displayed high potency in *AURKB *with an IC_50_ of 4 µM, 2 µM, and 3µM, compared to Barasertib IC_50_ of 0.7µM. Based on these results, 2p and 10a were more selective for *AURKA* while compound 2j exhibited greater selectivity for *AURKB,* as indicated by their respective IC_50_ and inhibition profile at10µM. These findings suggest that compounds 2p, 2j, and 10a have the potential to inhibit *AURK* activity. 

### Molecular docking

#### Molecular docking with AURKA (PDB ID: 3UP7)

**Figure 5:**
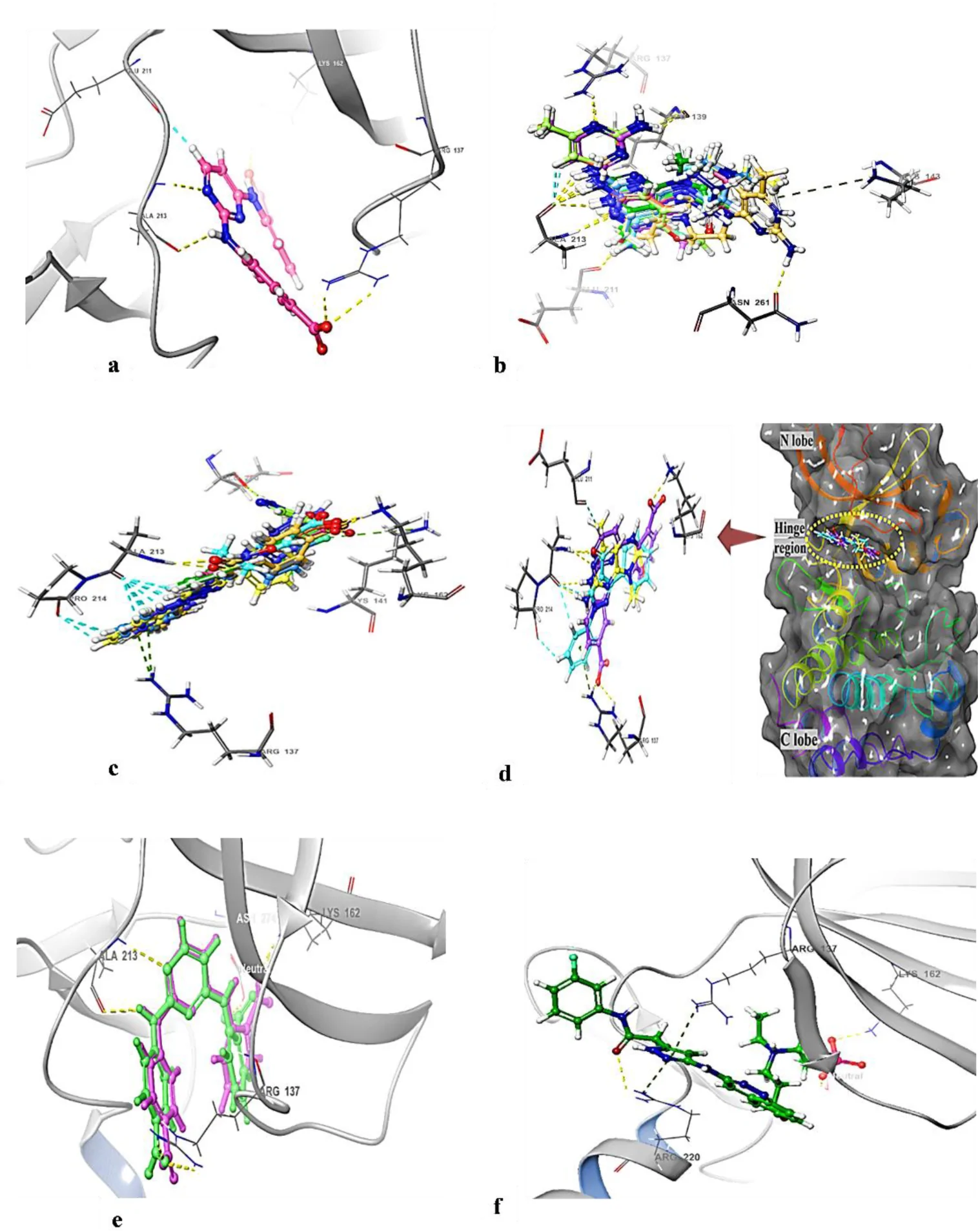
The binding interactions of ligands at the active site of the *AURKA* (PDB ID: 3UP7) a. Interactions of reference ligand; b. Interactions of the overlapped 2, 4-diaminopyrimidines; c. Interactions of the overlapped pyrazole-4-carbaldehydes and pyrazole-4-carboxylic acids; d. Interactions of the overlay of pyrazoles and 2,4-diaminopyrmidines along with the reference ligand (compound 2b; yellow color, compound 9a; blue color, compound 10 ligand; purple color); e. Validation of the molecular docking protocol for the receptor 3UP7. Green: Native ligand; Pink: Docked ligand; f. Interactions of Barasertib on *AURKA*.

The *AURKA* protein (PDB ID: 3UP7) was retrieved from protein data bank (PDB). This protein, derived from X-ray crystallography with a resolution of 3.07 A^0^. To validate the docking methodology, the Root Mean Square Deviation (RMSD) value was calculated, which indicated an accuracy of 0.969. The ATP binding site of AURKA is a complex region composed of several sub-pockets, with the hinge region playing a crucial role in ligand binding. Key amino acids within this region, such as Glu211, Ala213, Leu139, Leu263, Val147, and Lys162, were identified as potential interaction sites. The glide module within the maestro software was utilized for this analysis. To optimize the overall energy of the protein, a series of steps, including water molecule removal and polar hydrogen addition, were carried out using the protein preparation wizard. Subsequently, mol files were generated using the Maestro 2D sketcher tool. The LigPrep ligand processing tool was employed to process the mol files of the compounds, resulting in the generation of energy-minimized 3D molecular structures. To facilitate the subsequent docking study, a search grid specific to the 3UP7 protein structure was generated using Maestro's Glide grid generating tool. This search grid was designed to accommodate the reference ligand. Subsequently, the docking score in terms of kcal/mol and the interactions between all molecules were thoroughly analysed. The docking score for the reference ligand at 3UP7, namely 2-({2-[(4-carboxyphenyl) amino] pyrimidin-4-yl} amino) benzoic acid, was determined to be -9.7 kcal/mol. Various interactions, including hydrogen bonding and aromatic hydrogen bonding, were observed with various amino acids such as Ala213, Lys162, Arg137, and Glu211 (Figure 5(Fig. 5)). The synthesized compounds including 2,4-diaminopyrimidines, pyrazole-4-carbaldehydes, and pyrazole-4-carboxylic acids successfully occupy the ATP binding site of *AURKA* through hydrophobic interactions with neighboring amino acid residues (Figure 5(Fig. 5)), and this binding is further reinforced by a hydrogen bonding network involving the hinge backbone Ala213 and Glu211. Table 3(Tab. 3) presents the docking scores for 2,4-diaminopyrimdines, ranging from -7.8 to -3.8 kcal/mol and for pyrazole-4-carboxylic acids and pyrazole-4-carbaldehydes range from -7.6 to 5.5 kcal/mol. These scores, combined with the observed interactions, suggest that these compounds have the potential to inhibit *AURKA* and suppress cancer cell proliferation. A comparison with the known *AURKA *inhibitor, Barasertib, revealed similar binding modes, with scaffolds series forming critical interactions with Arg220, Arg137, and Lys162 residues in* AURKA*. This further supports the potential of the synthesized compounds as* AURKA* inhibitors. 

#### Molecular docking with AURKB (PDB ID: 4AF3)

**Figure 6:**
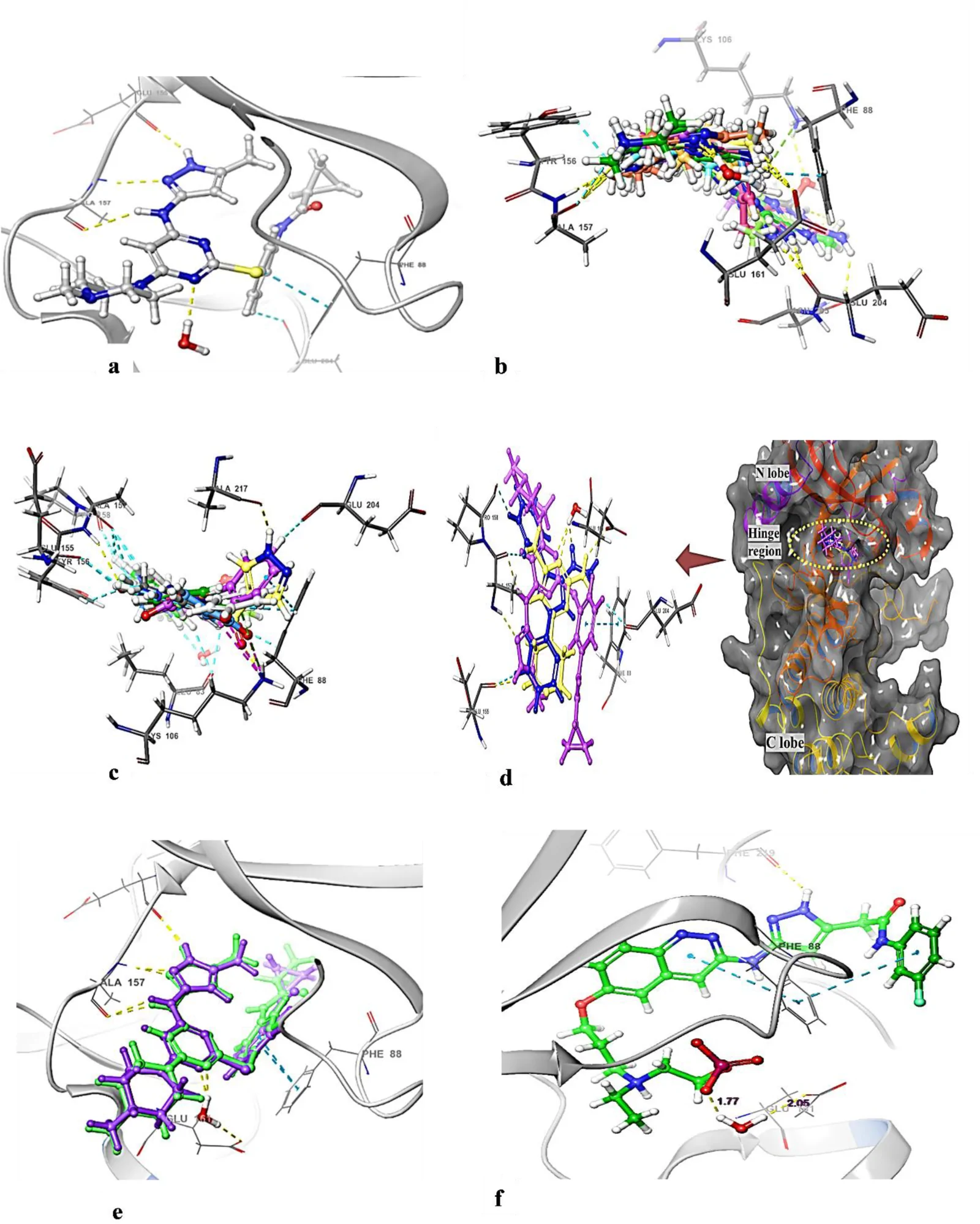
The binding interactions of ligands at the active site of the *AURKB* (PDB ID: 4AF3) a. Interactions of reference ligand; b. Interactions of the overlapped 2, 4-diaminopyrimidines; c. Interactions of the overlapped pyrazole-4-carbaldehydes and pyrazole-4-carboxylic acids; d. Interactions of the overlay of pyrazoles and 2,4-diaminopyrmidines along with the reference ligand (compound 2b; yellow color, compound 9a; blue color, compound 10 ligand; purple color); e. Validation of the molecular docking protocol for the receptor 4AF3. Green: Native ligand; Pink: Docked ligand; f. Interactions of Barasertib on *AURKB*.

To investigate the binding interactions of synthesized compounds with *AURKB* kinase, a molecular docking study was conducted using the X-ray crystal structure of *AURKB *(PDB ID: 4AF3, 2.75 A^0^), The protein was prepared for docking by removing water molecules and adding polar hydrogens, followed by energy minimization. The synthesized compounds were prepared using Maestro's 2D sketcher tool and energy minimized with LigPrep. The docking methodology was validated by re-docking a co-crystallized ligand, resulting in an RMSD 0.679, indicating accurate predictions. The docking study revealed that the synthesized compounds primarily interact with key amino acids in the hinge region of the *AURKB* kinase active site, including Ala157, Lys106, Phe88, Glu155, Pro158, Leu83, Glu161, and Glu204. Key interactions including hydrogen bonding between 2,4-diaminopyrimidines and Ala157, Glu155, and Lys106; hydrophobic interactions between Leu83 and Phe88 and the phenyl ring/carbonyl group of pyrzole-4-carboxylic acids/ pyrazole-4-carbaldehydes and π-π interactions with crucial amino acids in the ATP binding site (Figure 6(Fig. 6)). The docking score for 2, 4-diaminopyrimidines, ranged from -7.8 to -4.1 kcal/mol and for pyrazole-4-carboxylic acids and pyrazole-4-carbaldehydes, they ranged from -7.3 to 5.2 kcal/mol. The reference ligand had a docking score of -9.7 kcal/mol, and formed multiple interactions with key amino acids (Table 3(Tab. 3)). A molecular docking study with Barasertib revealed similar binding interactions, suggesting a conserved binding mode. 

### Predictions of molecular properties and drug likeliness 

The ADME (absorption, distribution, metabolism and excretion) properties of the synthesized molecules were calculated using QikProp tool, Schrodinger. This QikProp predicts physically significant descriptors and pharmaceutical relevant properties of the molecules. This highlighted some of the critical parameters like bioavailability and aqueous solubility of the compounds. Table 4(Tab. 4) lists some of the important ADME properties of the molecule. The metric QPlogS (Predicted Aqueous Solubility) forecasts a compound's solubility in water. A number between -6.5 and 0.5 is regarded as acceptable, with higher positive values denoting more solubility. The measure QPPCaCo (Predicted Apparent Caco-2 Cell Permeability), which simulates the gut-blood barrier, forecasts the apparent permeability of a substance across Caco-2 cell monolayers. While readings below 25 nm/sec represent poor permeability, values over 500 nm/sec suggest high permeability. Percentage of Human Oral Absorption: This measure calculates the proportion of a substance that enters the circulation after being administered orally. Less than 25 % is deemed bad, whereas more than 80 % is deemed high. A compound's binding affinity to human serum albumin is predicted using the QPlogKhsa (Prediction of Binding to Human Serum Albumin) formula. A reading between -1.0 and 1.5 is regarded as appropriate. A compound's capacity to pass the blood-brain barrier and enter the brain is predicted using the QLogBB (Prediction of Brain/Blood Partition Coefficient) formula. A number between -3.0 and 1.2 is regarded as acceptable. The molecule's mass is indicated by its molecular weight. For drug-like molecules, it should typically be fewer than 500 Da (Daltons). HBD (Hydrogen Bond Donor): This tally of the molecule's hydrogen bond donor groups. Generally, it ought to be lower than 5. Hydrogen Bond Acceptor (HBA): This tally of the molecule's hydrogen bond acceptor groups. Typically, it ought to be lower than 10. The predicted partition coefficient of the chemical between octanol and water is given by QPlogP(o/w) (Predicted Octanol/Water Partition Coefficient LogP). Higher numbers indicate more hydrophobicity, with an acceptable range being between -2.0 and 6.5. These criteria are used to evaluate a compound's potential pharmacological characteristics and drug-likeness. They assist in the screening and prioritisation of chemical compounds for subsequent drug discovery and development. As evident from the Table 4(Tab. 4), the values of the selected properties were well within range, and the molecules shows excellent oral absorption.

See also the Supplementary data.

## Conclusion

This study successfully designed, synthesized, and characterized small molecule inhibitors specifically 2, 4-diaminopyrimidines and pyrazoles analogues, targeting the key cell cycle regulators *AURKA *and *AURKB*. The compounds, prepared using both conventional and microwave methods, were confirmed for purity by UFLC and their structures definitively elucidated using FTIR, MS, and NMR (^1^H & ^13^C) spectroscopy. Critically these compounds showed favorable binding interaction within the hinge region of the target kinases: for *AURKA*, forming diverse bonds with residues including Ala213, Glu211, Leu139, and Lys162; and for *AURKB, *engaging Ala157, Glu155, Lys106 via hydrophilic bonds, alongside hydrophobic contributions from Leu83 and Phe88. Subsequently, the analogues were evaluated for cytotoxic activity on human SKOV3 (ovarian cancer), THP1 (leukemia), and C6 (brain cancer) cell lines using the MTT assay. 

Among synthesized compounds, 2k, 2j, 2l, 2m, 2p, 9a, and 10a exhibited dose-dependent cytotoxic activity across SKOV3, THP1 and C6 cancer cell lines. Notably, 2l and 2m demonstrated concentration-dependent cytotoxic activity with IC_50_ values of 8.63±0.05 and 62.57±13.220 µM respectively in SKOV3 cell line. Compounds 2k, 2l, 9a and 9b showed promising cytotoxic in a dose-dependent manner against C6 cell line with IC_50 _12.75±0.012, 0.49±0.001, 0.019±0.015, and 0.619±0.012 µM, respectively. For THP1, 2j, 2l, 2p, 9a, and 10a showed promising cytotoxic activity with IC_50_ 16.59±0.039, 0.18±0.026, 1.26±18.201, 0.64±56.358, and 0.084±0.030 µM respectively in dose-dependent manner. Based on cytotoxicity and their interactions at ATP active site of the kinases (3UP7, *AURKA*; 4AF3, *AURKB*) as confirmed by *in-silico* molecular docking, 2j, 2l, 2p, 9a, and 10a were further evaluated for the *AURKA* and 1 *AURKB* inhibition assays. Compounds 2p and 10a emerged as promising *AURKA* inhibitors, with the percentage inhibition of 68.14 and 55.64 % with IC_50_ 1 and 2 µM, respectively, relative to standard Barasertib which exhibited 78.42 % inhibition (IC_50_- 0.6 µM). Compounds 2j and 2p, showed 56.36 and 56.39 % inhibition of *AURKB* with IC_50_ 4 and 2 µM, respectively, compared to Barasertib with 68.62 % inhibition (IC_50_- 0.7 µM). respectively. In summary, this research successfully identified compounds 2p,10a, and 2j as potent *AURKA and AURKB *inhibitor. These molecules represent promising lead candidates for further development into effective, drug like inhibitors of *AURKA* and *AURKB*, paving the way for new chemical entities in cancer therapeutics.

## Experimental

### Chemistry

#### General

The solvents and reagents were procured from commercial sources such as S. D. Fine Chem (Mumbai, India), Sigma Aldrich (Germany), Loba Chem (Mumbai, India) All other solvents and reagents used were of analytical grade. The microwave synthesis of the compounds was conducted using CEM discover SP open vessel model. The synthesis progress was monitored by thin-layer chromatography (TLC) using Merck pre-coated Silica gel 60 F254 sheets (silica gel coated aluminium sheets obtained from Merck, specialities Ltd., Mumbai) and visualized under short UV (254 nm). The mobile phase was *n-*hexane: ethyl acetate (3:7) for 2,4-diaminopyrimidines and for pyrazole-4-carbaldehydes and pyrazole-4-carboxylic acids, the mobile phase was hexane: ethyl acetate (9:1). The intermediates were purified by recrystallization using appropriate solvents and final compounds were purified using column chromatography on silica gel 60 (#60-120) using a mixture of solvents, as mentioned in the synthetic procedures wherever applicable. The melting points of the synthesized compounds were measured using melting point equipment, VEEGO, VMP-D (General Trading Co., India) are expressed in ^o^C and are uncorrected. The Fourier transform infra-red (FTIR) spectrum was recorded using FTIR spectrophotometer (Shimadzu IR Affinity-IS) and expressed in cm^-1^. The scanning range used was 4000-400 cm^-1^. The purity of the synthesized compounds was determined using high performance liquid chromatography (HPLC). HPLC study was performed on Shimadzu, UFLC, LC-20AD lab solution software using a C18 Atlantis ® T3 column (25cm × 4.6mm, 5µ particle size) with methanol and isocratic mobile phase (A) methanol and (B) water in a 70:30 ratio (v/v) at a flow rate of 1mL/min. Deionized water was obtained from Millipore water (Milli-Q) purification system. The mass spectra (MS) were recorded on Shidmazu 8040 LC-MS/MS system with triple quadrupole (TQMS)(Shimadzu). The ^1^H and ^13^C-NMR spectra of the compounds were recorded on NMR spectrophotometer (500 MHz: Bruker Ascend 500) at Department of Chemistry, IIT Indore. Deuterated chloroform and DMSO was used as solvents. The splitting patterns are designated as follows: s, singlet; d, doublet; dd, doublet of doublet; m, multiplet. Chemical shifts values are expressed in δ parts per million (ppm).

#### Conventional method

0.002 mol of 2-amino-4-chloro-5-methyl-pyrimidines 1 was weighed and added to 100 mL round bottom flask; ethanol (30 mL) was added to it along with triethylamine (TEA) (0.002 mol) (Scheme 1 and 2). This reaction mixture was stirred till dissolution of solid material. Then substituted amines (-R_4_) in appropriate molar ratio were added to the reaction mixture (see Table 6) was stirred and heated at 70^0 ^C for 24-30 hrs. The completion of reaction was checked by TLC (*n*-hexane: ethyl acetate) (3:7). After cooling, the reaction mixture was poured into saturated sodium bicarbonate solution and was extracted with chloroform (×3). Anhydrous sodium sulphate was used for drying of organic extract, following filtration and concentrated under vacuum to obtain derivatives of 2-amino-4-chloro-5-methyl-pyrimidines (2a-2p)(Qureshi et al., 2022[9]).

#### Microwave method

0.002 mol of 2-amino-4-chloro-5-methyl-pyrimidines 1 was weighed and added to the 100 mL round bottom flask; ethanol (30 mL) was added to it along with triethylamine (TEA) (0.002 mol) (Scheme 1 and 2). This reaction mixture was stirred till dissolution of solid material. Then substituted amines (-R_4_) in appropriate molar ratio were added to the reaction mixture (Table 5(Tab. 5)). The reaction was subjected to microwave for 45-60 minutes at 130W at 70^0^C (CEM, Discover). The completion of reaction was checked by TLC (*n*-hexane: ethyl acetate). After cooling the reaction mixture poured into the saturated sodium bicarbonate water solution and extracted with chloroform (×3). Anhydrous sodium sulphate was used for drying of organic extract, following filtration and concentrated under vacuum to obtain derivatives of 2-amino-4-chloro-5-methyl-pyrimidines (2a-2p) (Scheme 1) (2a-2p).


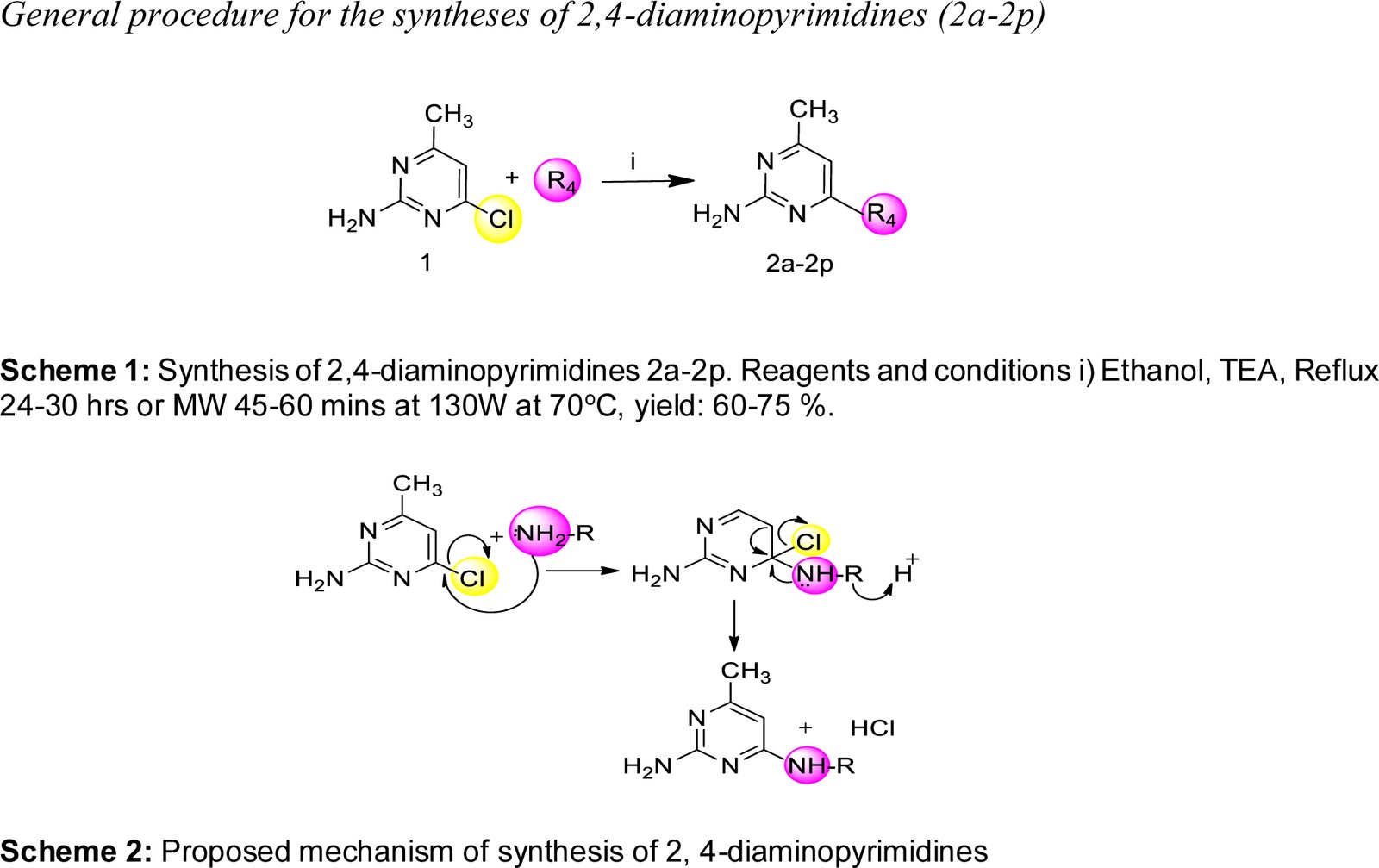


#### 4-methyl-6-morpholinopyrimidin-2-amine (2a)

2a was synthesized using 2-amino-4-chloro-5-methyl-pyrimidines 1 (0.002 mol), morpholine (0.002 mol), TEA (0.002 mol) as mentioned in the general procedure to yield 2a as off-white crystals. Yield: 70 %; TLC R*hf* = 0.20 (*n*-hexane: ethyl acetate, 3:7); purity (HPLC): 100 %; mp: 139-142^o^C; FTIR cm^-1^: 3396 (-NH stretch), 3129 (-CH, aromatic), 2914 (-CH, aliphatic), 1103 (-CN stretch); ^1^H NMR (500 MHz, CDCl_3_) δ (ppm): 5.79 (s, 1H, -CH), 4.785 (s, 2H, -NH_2_), 3.72 (t, 4H, J=5, -OCH_2_), 3.530 (t, 4H, J=4.5, -NCH_2_), 2.204 (s, 3H, -CH_3_); ^13^C NMR (500 MHz, CDCl_3_) δ (ppm): 166.64 (1C), 166.57(1C), 162.60 (1C), 92.74 (1C), 66.62 (2C), 44.17 (2C), 24.11 (1C); MS (ESI): *m/z*195 [m+1]^+^.

#### 4-methyl-6-(piperidin-1-yl) pyrimidin-2-amine (2b)

2b was synthesized using 2-amino-4-chloro-5-methyl-pyrimidines 1 (0.002 mol), piperidine (0.002 mol), TEA (0.002 mol) as mentioned in the general procedure to yield 2b as white crystals. Yield: 65 0 %; TLC R*f* = 0.22 (*n*-hexane: ethyl acetate, 3:7); purity (HPLC): 100 %; mp: 122-126^ o^C; FTIR cm^-1^: 3315 (-NH stretch), 3196 (-CH stretch, aromatic), 2929 (-CH stretch, aliphatic), 1022 (-CN stretch) ; ^1^H NMR (500 MHz, CDCl_3_) δ (ppm): 6.54 (s, 2H, NH_2_), 5.82 (s, 1H, -CH), 3.54 (m, 4H, -CH_2_), 2.32 (s, 3H, -CH_3_), 1.65 (m, 2H, CH_2_), 1.56 (m, 4H, -CH_2_); ^13^C NMR (500 MHz, CDCl_3_) δ (ppm): 165.86 (1C), 163.25 (1C), 162.65 (1C), 92.83 (1C), 44.95 (2C), 25.57 (2C), 24.77 (1C), 24.02 (1C); MS (ESI): *m/z*193.30 [m+1]^+^. 

#### 4-methyl-6-(pyrrolidin-1-yl) pyrimidin-2-amine (2c)

2c was synthesized using 2-amino-4-chloro-5-methyl-pyrimidines 1 (0.002mol), pyrrolidine (0.002mol), TEA (0.002 mol) as mentioned in the general procedure to yield 2c as yellow crystals. Yield: 70 %; TLC R*f* = 0.19 (*n*-hexane: ethyl acetate, 3:7); purity (HPLC): 100 %; mp: 128-133ºC ; FTIR cm^-1^: 3315 (-NH stretch), 3201 (-CH stretch, aromatic), 2920 (-CH stretch, aliphatic), 1579 (C=C Stretch), 1078 (CN stretch); ^1^H NMR (500 MHz, CDCl_3_) δ (ppm): 6.52 (s, 2H, -NH_2_), 5.61 (s, 1H, -CH), 3.63 (m, 4H, -CH_2_), 3.40 (s, 3H, -CH_3_), 2.62 (m, 4H, -CH_2_); ^13^C NMR (500 MHz, CDCl_3_) δ (ppm): 164.88 (1C), 162.49 (1C), 161.53 (1C), 93.76 (1C), 46.15 (2C), 25.23 (2C), 23.77; MS (ESI): *m/z *179.25 [m+1]^+^.

#### (N4-(4-fluorophenyl)-6-methylpyrimidine-2,4-diamine (2d)

2d*1* was synthesized using 2-amino-4-chloro-5-methyl-pyrimidines 1 (0.002mol), 4-fluoroaniline (0.002mol), TEA (0.002 mol) as mentioned in the general procedure to yield 2d as brown crystals. Yield: 72 %; TLC R*f* = 0.20 (*n*-hexane: ethyl acetate, 3:7); purity (HPLC): 74 %; mp: 136-138^ o^C; FTIR cm^-1^: 3307 (-NH), 3199 (-CH, aromatic), 2924 (-CH stretch), 1546 (C=C Stretch), 1635 (C=N stretch); ^1^H NMR (500 MHz, cdcl_3_) δ (ppm): 7.19 (m, 2H, Aromatic -CH), 6.97 (m, 2H, Aromatic -CH), 6.45 (s, 2H, -NH_2_), 5.76 (s, 1H, -CH), 4.87 (s, 1H, -NH), 2.23 (s, 3H, -CH_3_); ^13^C NMR (500 MHz, cdcl_3_) δ (ppm): 169.95 (1C), 167.08(1C), 162.69(1C), 161.33(1C), 134.48(1C), 124.99(2C), 110.15(2C), 93.83(1C), 23.64(1C); MS (ESI): *m/z *219.25 [m+1]^+^. 

#### 240 2-(4-(2-amino-6-methylpyrimidin-4-yl) piperazin-1-yl) ethanol (2e)

2e was synthesized using 2-amino-4-chloro-5-methyl-pyrimidines 1 (0.002 mol), N-(2-hydroxy-ethyl) piperazine (0.002 mol), TEA (0.002 mol) as mentioned in the general procedure to yield 2e as white crystals. Yield: 68 %; TLC R*f* = 0.22 (*n*-hexane: ethyl acetate, 3:7); purity (HPLC): 100 %; mp: 147-150ºC; FTIR cm^-1^: 3363 (-NH stretch), 3307 (-CH stretch), 2932 (-CH stretch aliphatic); ^1^H NMR (500 MHz, CDCl_3_) δ (ppm): 5.83 (s, 1H, -CH), 3.57 (t, 2H, J=5, CH2), 3.62 (tt, 4H, j=5, -CH2), 4.76 (s, 2H, -NH_2_), 2.62 (t, 2H, J=5, CH_2_), 2.19 (s, 3H, -CH_3_), 2.54 (tt, 4H, J=5, -CH_2_);^13^C NMR (500 MHz, CDCl_3_) δ (ppm): 166.19 (1C), 163.40(1C), 162.41(1C), 92.95(1C), 59.54(1C), 57.81(1C), 52.62(2C), 43.81(2C), 23.96(1C); MS (ESI): *m/z *238.25 [m+1]^+^. 

#### N4, N4-diethyl-6-methylpyrimidine-2,4-diamine (2f)

2f was synthesized using 2-amino-4-chloro-5-methyl-pyrimidines 1 (0.002mol), diethylamine (0.015mol), TEA (0.002 mol) as mentioned in the general procedure to yield 2f as yellow crystals. Yield: 75 %; TLC R*f* = 0.19 (*n*-hexane: ethyl acetate, 3:7); purity (HPLC): 95 %; mp: 108-110ºC; FTIR cm^-1^: 3390 (-NH stretch), 2362 (-CH aliphatic), 2920 (-CH stretch), 1637 (C=N stretch); ^1^H NMR (500 MHz, CDCl_3_) δ (ppm): 5.63 (s, 1H, -CH), 4.81 (s, 2H, -NH_2_), 3.36 (q, 4H, J=7.1, CH_2_), 2.23 (s, 3H, -CH_3_), 1.08 (t, 6H, J=7.1, CH_3_).; ^13^C NMR (500 MHz, CDCl_3_) δ (ppm): 169.88 (1C), 165.01(1C), 162.80(1C), 92.48(1C), 41.74(2C), 23.62(1C), 12.90(2C); MS (ESI): *m/z *181.25 [m+1]^+^. 

#### N4, N4,6-trimethylpyrimidine-2,4-diamine (2g)

2g was synthesized using 2-amino-4-chloro-5-methyl-pyrimidines 1 (0.002mol), dimethylamine (0.007 mol), TEA (0.002 mol) as mentioned in the general procedure to yield 2g as off-white crystals. Yield: 65 %; TLC R*f* = 0.20 (*n*-hexane: ethyl acetate, 3:7); purity (HPLC): 100 %; mp: 122-126ºC; FTIR cm^-1^: 3367 (-NH stretch), 2334 (-CH aliphatic), 2922 (-CH stretch), 1639 (C=N stretch); ^1^H NMR (500 MHz, CDCl_3_) δ (ppm): 5.65 (s, 1H,-CH), 4.89 (s, 2H, -NH_2_), 2.94 (s, 6H, -CH_3_), 2.12 (s, 3H, -CH_3_); ^13^C NMR (500 MHz, CDCl_3_) δ (ppm): 165.25 (1C), 163.63(1C), 162.35(1C), 92.56(1C), 37.02 (2C), 23.78(1C); MS (ESI): *m/z *152.11 [m+1]^+^. 

#### N4-(4-methoxyphenyl)-6-methylpyrimidine-2,4-diamine (2h)

2h was synthesized using 2-amino-4-chloro-5-methyl-pyrimidines 1 (0.002mol), 4-methoxyaniline (0.008 mol), TEA (0.002 mol) as mentioned in the general procedure to yield 2h as brown crystals. Yield: 68 %; TLC R*f* = 0.19 (*n*-hexane: ethyl acetate, 3:7); purity (HPLC): 96 %; mp: 218-220ºC; FTIR cm^-1^: 3550 (-NH), 3383 (-CH stretch), 2924 (-CH stretch, aliphatic), 1533 (C=C stretch); ^1^H NMR (500 MHz, CDCl_3_) δ (ppm): 6.64 (m, 2H, Aromatic -CH), 6.53 (m, 2H, Aromatic -CH), 6.78 (s, 2H, -NH_2_), 5.68 (s, 1H, -CH), 4.88 (s, 1H, -NH), 3.63 (s, 3H, OCH_3_), 3.28 (s, 3H, CH_3_); ^13^C NMR (500 MHz, CDCl_3_) δ (ppm): 163.06 (1C), 157.05(1C), 152.73(1C), 147.27(1C), 140.04 (1C), 117.01(2C), 114.18 (2C), 93.75(1C), 55.87(1C), 29.73(1C); MS (ESI): *m/z *231.30 [m+1]^+^. 

#### 6-methyl-N4-(p-tolyl) pyrimidine-2,4-diamine (2i)

2i was synthesized using 2-amino-4-chloro-5-methyl-pyrimidines 1 (0.002mol), 4-methylaniline (0.002 mol), TEA (0.002 mol) as mentioned in the general procedure to yield 2i as orange crystals. Yield: 79 %; TLC R*f* = 0.19 (*n*-hexane: ethyl acetate, 3:7); purity (HPLC): 95 %; mp: 218-220ºC; FTIR cm^-1^: 3456 (-NH stretch), 3300 (-CH stretch, aromatic), 2918 (-CH, aliphatic), 1269 (-CN stretch); ^1^H NMR (500 MHz, CDCl_3_) δ (ppm): 7.17 (d, 4H, J=1.9, Aromatic -CH), 6.53 (s, 2H, -NH_2_), 5.94 (s, 1H, -CH), 4.76 (s, 1H, -NH), 2.35 (s, 3H, -CH_3_), 2.19 (s, 3H, -CH_3_); ^13^C NMR (500 MHz, CDCl_3_): δ (ppm): 167.29 (1C), 162.77 (1C), 162.54 (1C), 135.83 (1C), 134.42 (1C), 129.87 (2C), 123.01 (2C), 93.96 (1C), 24.01 (1C), 20.91 (1C); MS (ESI): *m/z *215.20 [m+1]^+^. 

#### 4-(4-ethylpiperazin-1-yl)-6-methylpyrimidin-2-amine (2j)

2j was synthesized using 2-amino-4-chloro-5-methyl-pyrimidines 1 (0.002mol), 4-ethylpiperazine (0.002 mol), TEA (0.002mol) as mentioned in the general procedure to yield 2j as light brown crystals. Yield: 65 %; TLC R*f* = 0.20 (*n*-hexane: ethyl acetate, 3:7); purity (HPLC): 96 %; mp: 88-90ºC; FTIR cm^-1^: 3483 (-NH stretch), 3398 (-CH stretch, aromatic), 2920 (-CH, stretch), 1215 (C-N stretch), 2852 (-CH, stretch), 1544 (C=C stretch); ^1^H NMR (500 MHz, DMSO) δ (ppm): 1.03 (t, 3H, J=5, -CH_3_), 2.30 (m, 2H, -CH _2_), 3.48 (t, 4H, J=5, -CH_2_), 5.90 (s, 1H), 7.02 (s, 2H, NH_2_), 2.07 (s, 3H, CH_3_), 3.35 (m, 4H, -CH_2_);^13^C NMR (500 MHz, DMSO) δ (ppm): 166.00 (1C), 163.57 (1C), 160.39 (1C), 108.50 (1C), 91.99 (1C), 52.60 (1C), 52.12 (1C), 43.79 (1C), 40.39 (1C), 40.14 (1C), 24.08 (1C), 12.41 (1C); MS (ESI): *m/z *222.30[m+1]^+^. 

#### 4-methyl-6-(4-methylpiperazin-1-yl) pyrimidin-2-amine (2k)

2k was synthesized using 2-amino-4-chloro-5-methyl-pyrimidines 1 (0.002 mol), 4-methylpiperazine (0.002 mol), TEA (0.002 mol) as mentioned in the general procedure to yield 2k as yellow crystals. Yield: 70 %; TLC R*f* = 0.23 (*n*-hexane: ethyl acetate, 3:7); purity (HPLC): 91 %; mp: 140-142ºC; FTIR cm^-1^: 3402 (-NH stretch), 3184 (-CH stretch, aromatic), 2920 (-CH, stretch), 1220 (C-N stretch), 2798 (-CH, stretch); ^1^H NMR (500 MHz, DMSO) δ (ppm): 6.56 (s, 2H, -NH_2_), 5.96 (s, 1H, -CH), 3.57 (t, 4H, J=5.1, -CH_2_), 2.31 (t, 4H, J=5, -CH_2_), 2.19 (s, 3H, -CH_3_), 2.08 (s, 3H, -CH_3_); ^13^C NMR (500 MHz, CDCl_3_) δ (ppm): 165.99 (1C), 163.55 (1C), 163.18 (1C), 92.03 (1C), 59.62 (1C), 57.60 (1C), 54.79 (1C), 46.23(1C), 43.67 (1C), 23.69 (1C); MS (ESI): *m/z *208.20[m+1]^+^. 

#### 4-methyl-6-(4-phenylpiperazin-1-yl) pyrimidin-2-amine (2l)

2l was synthesized using 2-amino-4-chloro-5-methyl-pyrimidines 1 (0.002 mol), 4-phenylpiperazine (0.003mol), TEA (0.002 mol) as mentioned in the general procedure to yield 2l as white crystals. Yield: 76 %; TLC R*f* = 0.17 (*n*-hexane: ethyl acetate, 3:7); purity (HPLC): 86 %; mp: 202-204ºC; FTIR cm^-1^: 3481 (-NH stretch), 3294 (-CH stretch, aromatic), 2918 (-CH stretch, aliphatic), 1583 (C=C stretch), 1165 (-CN stretch); ^1^H NMR (500 MHz, cdcl_3_) δ (ppm): 7.25 - 7.17 (m, 2H, Aromatic -CH), 6.82 (tt, 1H, J=7.2, 1.1, 1, Aromatic -CH), 6.88 (dt, 2H, J=7.8, Aromatic -CH), 5.80 (s, 1H, -CH), 4.73 (s, 2H, -NH_2_), 3.67 (t, 4H, J=5, -CH_2_), 3.15 (t, 4H, J=5, -CH_2_), 2.16 (s, 3H, CH_3_); ^13^C NMR (500 MHz, cdcl_3_) δ (ppm): 166.60 (1C), 163.45(1C), 162.63(1C), 151.11(1C), 129.27(2C), 120.23(1C), 116.39(2C), 93.02(1C), 49.12(2C), 43.73(2C), 24.18(1C).MS (ESI): *m/z *270.25[m+1]^+^. 

#### N4-benzyl-6-methylpyrimidine-2,4-diamine (2m)

2m was synthesized using 2-amino-4-chloro-5-methyl-pyrimidines 1 (0.002 mol), benzylamine (0.012 mol), TEA (0.002 mol) as mentioned in the general procedure to yield 2m as orange crystals. Yield: 65 %; TLC R*f* = 0.23 (*n*-hexane: ethyl acetate, 3:7); purity (HPLC): 88 %; FTIR cm^-1^: 3477 (-NH stretch), 3253 (-CH stretch, aromatic), 2988 (-CH stretch, aliphatic), 1572 (C=C stretch); ^1^H NMR (500 MHz, cdcl_3_) δ (ppm): 7.33 - 7.24 (m, 5H, Aromatic -CH), 6.34 (s, 2H, -NH_2_), 5.48 (s, 1H, -CH), 3.50 (t, 2H, J=5, -CH_2_), 4.33 (s, 1H, -NH), 2.01 (s, 3H, -CH_3_); ^13^C NMR (500 MHz, cdcl_3_) δ (ppm): 169.87 (1C), 163.92(1C), 162.61(1C), 138.52(1C), 128.71(2C), 128.32(2C), 127.18(1C), 109.86, (1C), 45.09(1C), 23.53(1C); MS (ESI): *m/z *215.25[m+1]^+^. 

#### 4-methyl-6-(piperazin-1-yl) pyrimidin-2-amine (2n)

2n was synthesized using 2-amino-4-chloro-5-methyl-pyrimidines 1 (0.002 mol), piperazine (0.002 mol), TEA (0.002 mol) as mentioned in the general procedure to yield 2n as white crystals. Yield: 70 %; TLC R*f* = 0.20 (*n*-hexane: ethyl acetate, 3:7); purity (HPLC): 100 %; mp:201-203 ºC; FTIR cm^-1^: 3339 (-NH stretch), 3149 (-CH stretch, aromatic), 2921 (-CH, stretch, aliphatic), 1223 (C-N stretch); ^1^H NMR (500 MHz, cdcl_3_) δ (ppm): 5.73 (s, 1H, -CH), 4.90 (s, 2H, -NH_2_), 3.57 (dt, 4H. J=5,5, -CH_2_), 2.83 (dt, 4H, J=5,5, -CH_2_), 2.12 (s, 3H, -CH_3_), 1.93 (s, 1H, -NH); ^13^C NMR (126 MHz, cdcl_3_) δ (ppm): 165.96 (1C), 163.49(1C), 162.37(1C), 92.82(1C), 59.36(1C), 56.79(1C), 45.76(1C), 44.88(1C), 23.91(1C); MS (ESI): *m/z *194.25[m+1]^+^. 

#### N4-ethyl-6-methylpyrimidine-2,4-diamine (2o)

2o was synthesized using 2-amino-4-chloro-5-methyl-pyrimidines 1 (0.002 mol), ethylamine (0.008 mol), TEA (0.002 mol) as mentioned in the general procedure to yield 2o as yellow crystals. Yield: 65 %; TLC R*f* = 0.19 (*n*-hexane: ethyl acetate, 3:7); purity (HPLC): 98 %; mp: 120-128 ºC; FTIR cm^-1^: 3315 (-NH stretch), 3187 (-CH stretch, aromatic), 2853 (-CH, stretch, aliphatic); ^1^H NMR (500 MHz, CDCl_3_) δ (ppm): 6.55 (s, 2H, -NH_2_), 5.38 (s, 1H, -CH), 1.23 (m, 3H, CH_3_), 4.86 (s, 1H,-NH), 2.20 (s, 3H, CH_3_), 3.67 (q, 2H, J=6.5, CH2); ^13^C NMR (126 MHz, CDCl_3_) δ (ppm): δ 163.78 (2C), 162.58 (1C), 110.39 (1C), 36.09 (1C), 23.76 (1C), 14.75 (1C); MS (ESI): *m/z *153.25[m+1]^+^. 

#### 6-methyl-N4-propylpyrimidine-2,4-diamine (2p)

2p was synthesized using 2-amino-4-chloro-5-methyl-pyrimidines 1 (0.002 mol), propylamine (0.01 mol), TEA (0.002 mol) as mentioned in the general procedure to yield 2p as yellow crystals. Yield: 68 %; TLC R*f* = 0.21 (*n*-hexane: ethyl acetate, 3:7); purity (HPLC): 93 %; FTIR cm^-1^: 3311 (-NH stretch), 3196 (-CH stretch, aromatic), 2963 (-CH, stretch, aliphatic); ^1^H NMR (500 MHz, CDCl_3_) δ (ppm): 6.55 (s, 2H, -NH_2_), 5.64 (s, 1H, -CH), 4.75 (s, 1H, -NH), 3.69 (t, 2H, J=2, 5, -CH_2_), 2.68 (s, 3H, CH_3_), 1.61 (m, 2H, -CH_2_), 0.96 (t, 3H, J=10, CH_3_); ^13^C NMR (500 MHz, CDCl_3_) δ (ppm): 166.19 (1C), 163.93 (1C), 162.55(1C), 56.70(1C), 43.15(1C), 23.74(1C), 22.71(1C), 11.42(1C); MS (ESI): *m/z *167.25[m+1]^+^.

#### General synthesis of 2-amino-6-methylpyrimidin-4-ol (5)

A guanidine hydrochloride 3 (0.052 mol) and ethyl acetoacetate 4 (0.052 mol) were placed in in water (15mL) (Scheme 3 and 4). To this solution, sodium carbonate (0.052 mol) was added and refluxed it at 100^0^C for 5 hrs to obtain final product 5 with 76 % yield. The completion of reaction was monitored by TLC (chloroform: methanol) (9.6:0.4) (Bayramoğlu et al., 2020[2]). 


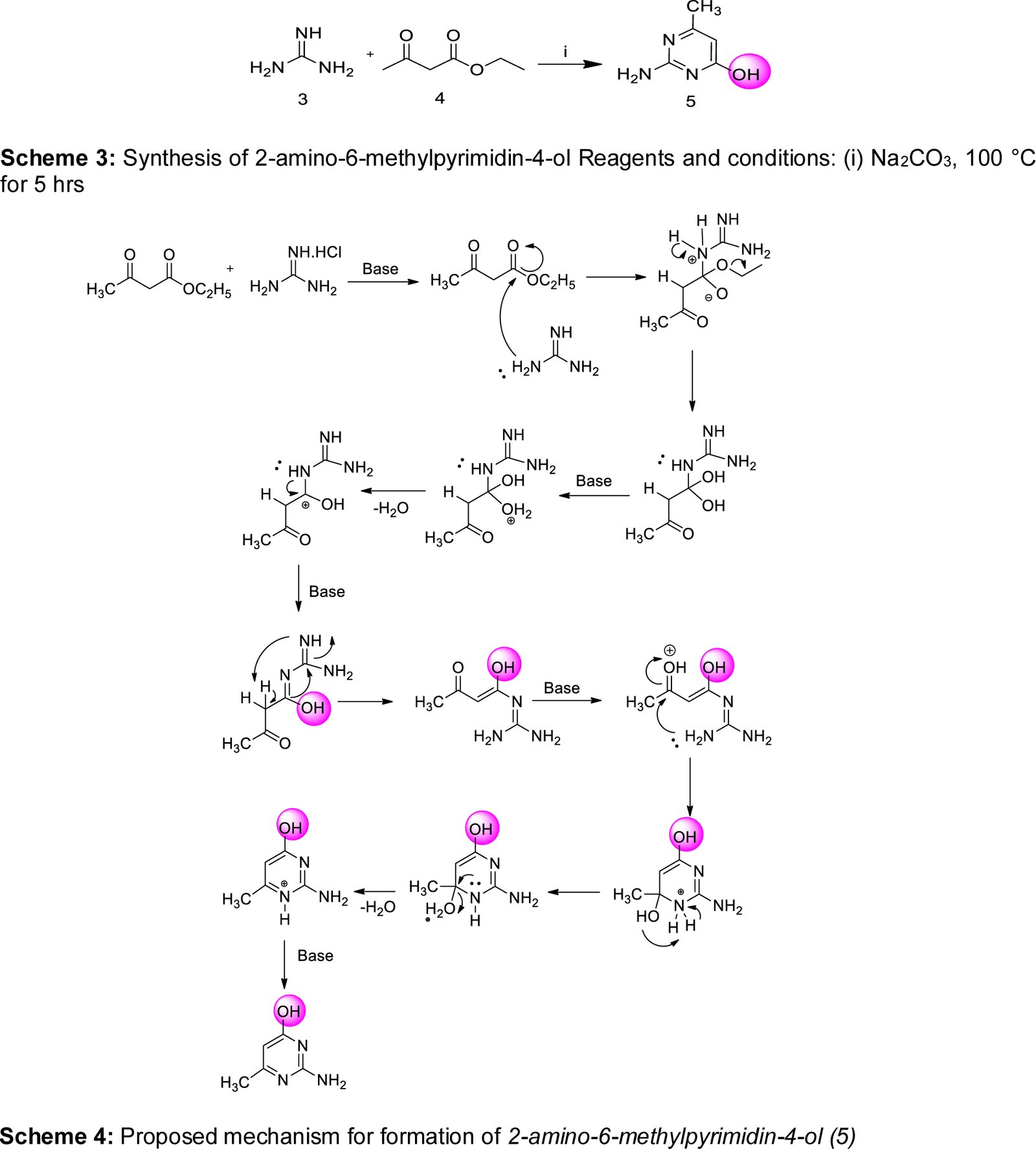


#### General procedure for Syntheses of pyrazole-4- carbaldehydes (9a-9e)

In a round bottom flask, acetophenone 6 (0.1 mol) and phenyl hydrazine 7 (0.1 mol) were added, and heated on a water bath for 60 minutes (Scheme 5 and 6). The reaction mixture was then poured in 30-40 mL of ethanol and vigorously stirred to obtain crystals of 8, vacuum-dried, and taken up right away for the next step. Acetophenone phenylhydrazones 8 (0.015 mol) was added to 25 mL cold solution of DMF (0.32 mol), followed by the addition of cold 5 mL of POCl_3 _(0.05 mol), and the mixture was stirred at 50-60 °C for 5 hours. To neutralize the mixture, a saturated solution of NaHCO_3_ was added (Table 6(Tab. 6)). The solid precipitate 9 was then filtered, washed with water, vacuum-dried and taken up for further reaction (Abdel-Wahab et al., 2011[1]).


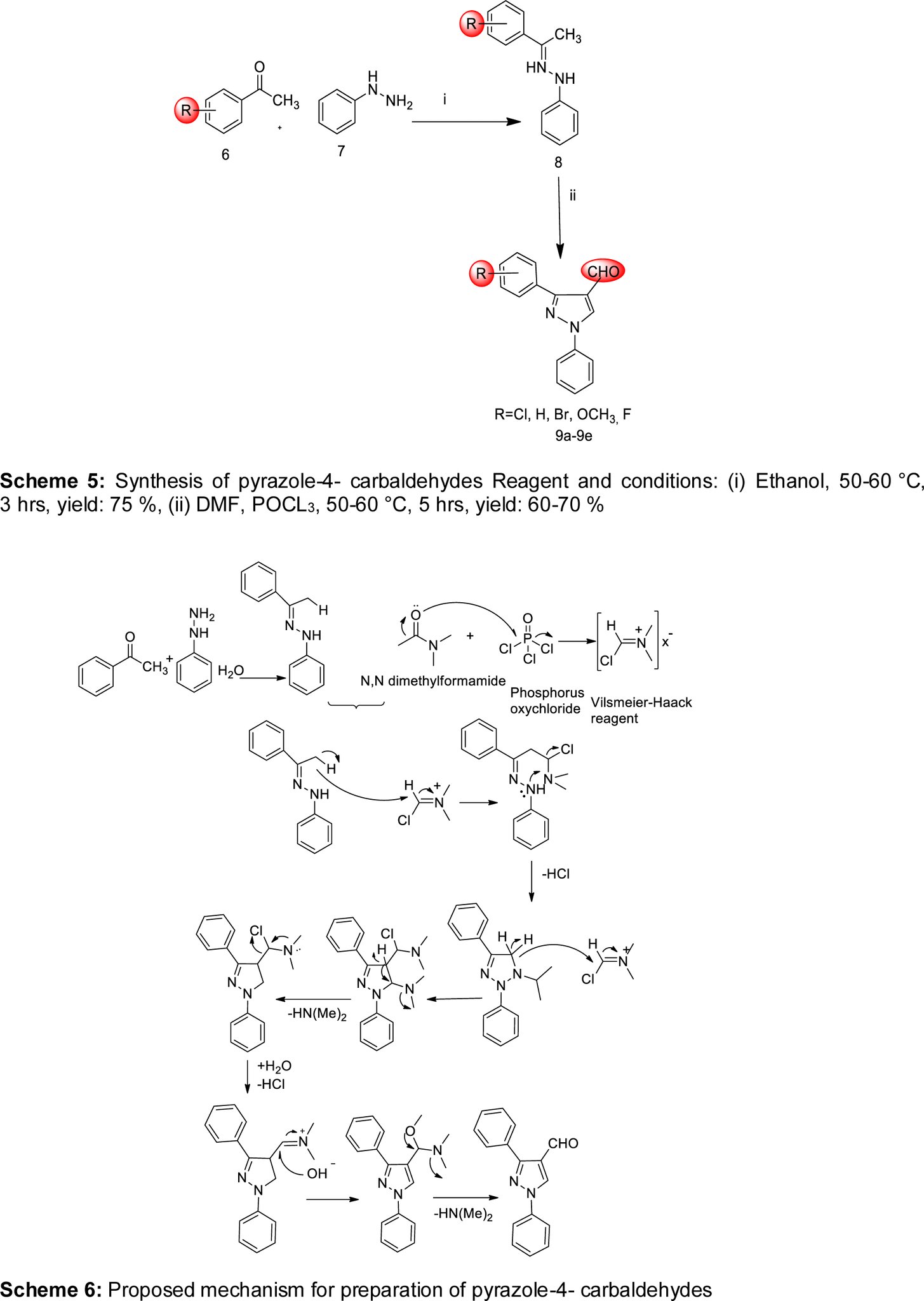


#### 1,3-diphenyl-H-pyrazole-4-carbaldehyde (9a)

9a was synthesized using Acetophenone phenylhydrazones 8 (0.015 mol), 25 mL cold solution of DMF (0.32 mol), 5 mL of POCl_3_ (0.05 mol), as mentioned in the general procedure to yield 9a as off-white crystals. Yield: 70 %; TLC R*f* = 0.35 (*n*-hexane: ethyl acetate, 9:1); purity (HPLC): 100 %; mp: 135-138ºC; FTIR cm^-1^: 3121 (C-H Stretch), 1510 (C=N), 1671 (C=O); ^1^H NMR (500 MHz, CDCl_3_) δ (ppm): 10.05 (s, 1H, -CHO), 8.55 (s, 1H, -CH), 7.51 (d, 2H, J=5, Aromatic -CH), 7.40 (d, 2H, J=6.9, Aromatic -CH), 7.34 (m, 3H, Aromatic -CH), 7.26 (m, 1H, Aromatic -CH), 7.82 (m, 2H, Aromatic -CH); ^13^C NMR (500 MHz, CDCl_3_) δ (ppm): 185.29 (1C), 154.86 (1C), 139.04 (1C), 133.29 (1C), 130.97 (1C), 129.86 (2C), 129.30 (2C), 128.86 (2C), 128.80 (1C), 128.64 (1C), 128.26 (1C), 119.80 (2C); MS (ESI): *m/z *249.30 [m+1]^+^. 

#### 3-(4-chlorophenyl)-1-phenyl-H-pyrazole-4-carbaldehyde (9b)

9b was synthesized using 4-chloroacetophenone phenylhydrazones 8 (0.015 mol), 25 mL cold solution of DMF (0.32 mol), 5 mL of POCl_3_ (0.05 mol), as mentioned in the general procedure to yield 9b as grey crystals. Yield: 60 %; TLC R*f* = 0.26 (*n*-hexane: ethyl acetate, 9:1); purity (HPLC): 95 %; mp: 135-138ºC; FTIR cm^-1^: 3121 (C-H Stretch), 834 (C-Cl), 1667 (C=O), 1515 (C=N); ^1^H NMR (500 MHz, CDCl_3_) δ (ppm): 10.04 (s, 1H, -CHO), 8.54 (s, 1H, -CH), 7.90-7.83 (m, 2H, Aromatic -CH), 7.79 (d, 2H, J=8.0, Aromatic -CH), 7.52 (t, 2H, J=7.8, Aromatic -CH), 7.41 (t, 1H, J=7.4, Aromatic -CH), 7.20 (t, 2H, J=8.6, Aromatic -CH); ^13^C NMR (500 MHz, CDCl_3_) δ (ppm): 184.49 (1C), 153.24 (1C), 138.92 (1C), 135.46 (1C), 132.02 (1C), 130.21 (1C), 129.86 (2C), 129.76 (1C), 128.96 (2C), 128.14 (1C), 122.53 (1C), 114 (1C), 119.77 (2C); MS (ESI): *m/z *283.30 [m+1]^+^.

#### 3-(4-fluorophenyl)-1-phenyl-H-pyrazole-4-carbaldehyde (9c)

9c was synthesized using 4-fluoroacetophenone phenylhydrazones 8 (0.015 mol), 25 mL cold solution of DMF (0.32 mol), 5 mL of POCl_3_ (0.05 mol), as mentioned in the general procedure to yield 9c as yellow crystals. Yield: 75 %; TLC R*f* = 0.29 (*n*-hexane: ethyl acetate, 9:1); purity (HPLC): 99 %; mp: 142-145ºC; FTIR cm^-1^: 3121 (C-H Stretch), 1666.64 (C=0), 1516 (C=N), 1222 (C-F); ^1^H NMR (500 MHz, CDCl_3_) δ (ppm): 10.03 (s, 1H, -CHO), 8.53 (s, 1H, -CH), 7.91-7.84 (m, 2H, Aromatic -CH), 7.78 (d, 2H, J=5, Aromatic -CH, 7.52 (t, 2H, J= 7.9, 2, Aromatic -CH), 7.46-7.37 (m, 1H, Aromatic -CH), 7.19 (t, 2H, J= 8.6, Aromatic -CH); ^13^C NMR (500 MHz, CDCl_3_) δ (ppm): 182.73 (1C), 162.60 (1C), 151.58 (1C), 137.04 (1C), 129.96 (2C), 128.95 (2C), 128.89 (1C), 127.83 (1C), 126.18 (1C), 120.53 (2C), 116.92 (2C), 114.49 (1C); MS (ESI): *m/z *267.30 [m+1]^+^.

#### 3-(4-bromophenyl)-1-phenyl-H-pyrazole-4-carbaldehyde (9d)

9d was synthesized using 4-bromoacetophenone phenylhydrazones 8 (0.015 mol), 25 mL cold solution of DMF (0.32 mol), 5 mL of POCl_3 _(0.05 mol), as mentioned in the general procedure to yield 9d as white crystals. Yield: 75 %; TLC R*f* = 0.30 (*n*-hexane: ethyl acetate, 9:1); purity(HPLC): 93 %; mp: 138-140ºC; FTIR cm^-1^: 3121 (C-H Stretch), 1515 (C=N), 1675 (C=0), 752 (Br);^ 1^H NMR (500 MHz, CDCl_3_) δ (ppm): 10.03 (s, 1H, -CHO), 8.53 (s, 1H, -CH), 7.77 (t, 4H, J=8.1, Aromatic -CH), 7.63 (d, 2H, J=8.3, Aromatic -CH), 7.52 (t, 2H, J=7.8, Aromatic -CH), 7.41 (t, 1H, J=7.4, Aromatic -CH); ^13^C NMR (500 MHz, CDCl_3_) δ (ppm): 187.18 (1C), 153.54 (1C), 142.26 (1C), 133.35 (1C), 131.36 (1C), 131.30 (1C), 130.31(2C), 121.11 (1C), 129.68 (2C), 127.81 (2C), 119.62 (2C), 114.94 (1C); MS (ESI): *m/z *327.25 [m+1]^+^.

#### 3-(4-methoxyphenyl)-1-phenyl-H-pyrazole-4-carbaldehyde (9e)

9e was synthesized using 4-methoxyoacetophenone phenylhydrazones 8 (0.015 mol), 25 mL cold solution of DMF (0.32 mol), 5 mL of POCl_3 _(0.05 mol), as mentioned in the general procedure to yield 9e as white crystals. Yield: 70 %; TLC R*f* = 0.40 (*n*-hexane: ethyl acetate, 9:1); purity (HPLC): 92 %; mp: 136-140ºC; FTIR cm^-1^: 3120 (C-H Stretch), 1515 (C=N), 1666 (C=0), 2838 (OCH_3_); ^1^H NMR (500 MHz, CDCl_3_) δ (ppm): 10.04 (s, 1H, -CHO), 8.52 (s, 1H, -CH), 7.03 (d, 2H, J=8.6, Aromatic -CH), 7.43-7.36 (m, 1H, Aromatic -CH), 7.51 (t, 2H, J=5, Aromatic -CH), 7.82-7.77 (m, 4H, Aromatic -CH), 3.88 (s, 3H); ^13^C NMR (500 MHz, CDCl_3_) δ (ppm): 185.23 (1C), 160.57 (1C), 154.55 (1C), 139.07 (1C), 131.18 (1C), 130.28 (1C), 129.69 (2C), 127.90 (2C), 123.86 (1C), 122.37 (1C), 119.74 (2C), 114.22 (2C), 55.41 (1C); MS (ESI): *m/z *279.35 [m+1]^+^.

#### General procedure for Syntheses of pyrazole-4- carboxylic acids

A sodium dihydrogen phosphate (NaH_2_PO_4_) (0.01 mol) and potassium permanganate (0.05 mol) in water were added to a solution of pyrazole carbaldehydes 9 (0.015mol) in acetone, and the reaction mixture was agitated for 2-3 hours at 50-60°C. The mixture changes from violet to brown. After removing the manganese dioxide, a solution of sodium metabisulphite was added dropwise until the color disappeared. The resultant mixture was placed in an ice bath with a few drops of concentrated HCl was added to it until the pH of the filtrate was 1 to 2. The resultant precipitate 10 was filtered, rinsed with cold water, dried, and recrystallized with ethanol (Table 7(Tab. 7)) (Scheme 7 and 8) (Bratenko et al., 2001[4]).


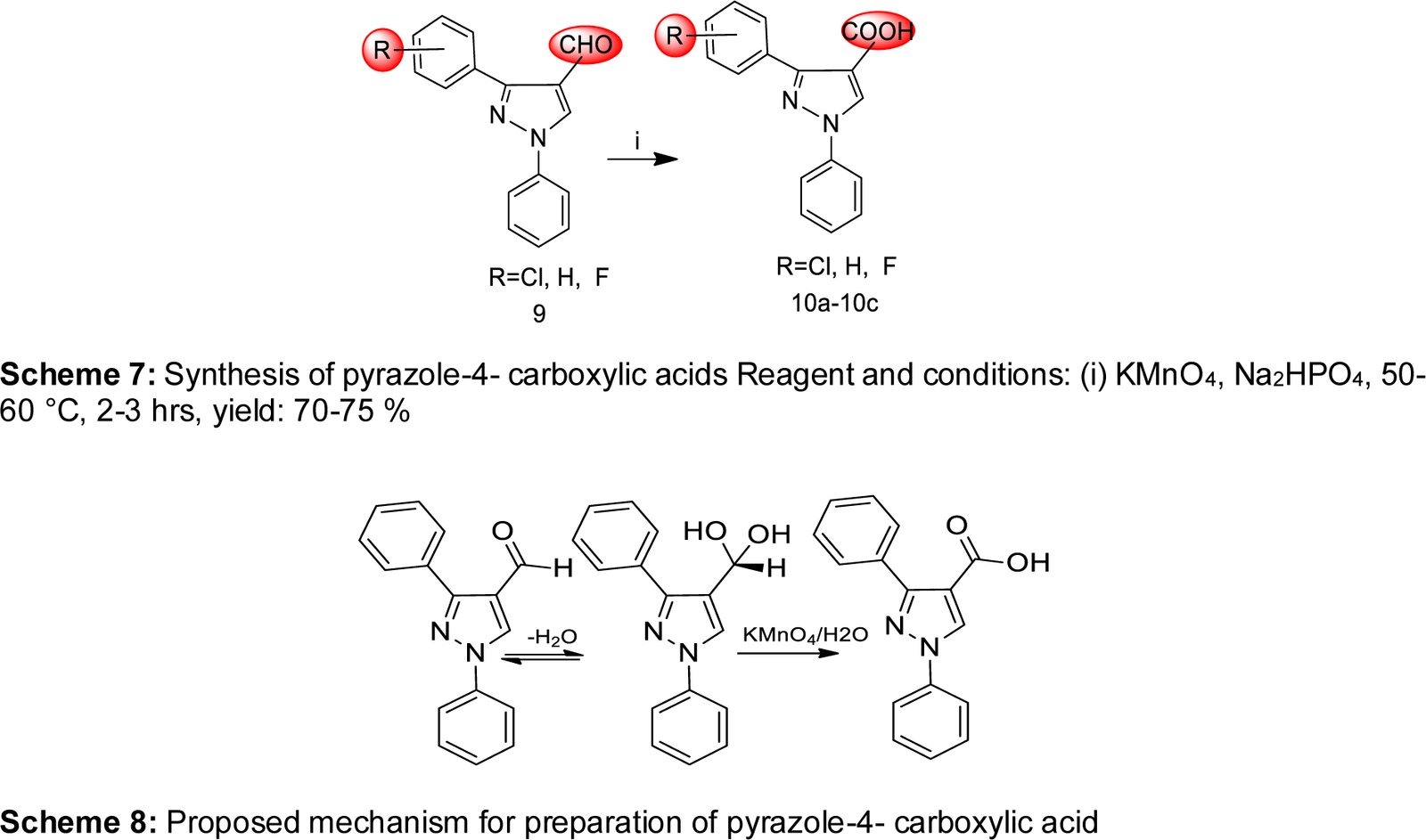


#### 1,3-diphenyl-H-pyrazole-4-carboxylic acid (10a)

10a was synthesized using Na_2_HPO_4_ (0.01 mol), potassium permanganate (0.05 mol), 9a (0.015mol) as mentioned in the general procedure to yield 10a as white crystals. Yield: 70 %; TLC R*f* = 0.45 (*n*-hexane: ethyl acetate, 6:4); purity (HPLC): 99 %; mp: 180-182^0^C; FTIR cm^-1^: 3428 (broad OH stretch), 1677 (C=O); ^1^H NMR (500 MHz, DMSO) δ (ppm): 8.588 (s, 1H,-CH), 7.881 (d, 2H, J=6, Aromatic -CH), 7.788 (d, 2H, J=7.5, Aromatic -CH), 7.506 (t, 2H, J=8, Aromatic -CH), 7.490-7.442 (m, 3H, Aromatic -CH), 7.381 (t, 1H, J=7, Aromatic -CH); ^13^C NMR (500 MHz, DMSO) δ (ppm): 169.14 (1C), 157.99(1C), 144.05(1C), 138.74(1C), 137.41(1C), 134.85(2C), 134.35(2C), 133.66(2C), 133.03(2C), 132.47(1C), 124.22(1C), 119.46(1C); MS (ESI): *m/z *265.15 [m+1]^+^.

#### 3-(4-chlorophenyl)-1-phenyl-H-pyrazole-4-carboxylic acid (10b)

10b was synthesized using Na_2_HPO_4 _(0.01 mol), potassium permanganate (0.05 mol), 9b (0.015mol) as mentioned in the general procedure to yield 10b as off-white crystals. Yield: 78 %; TLC R*f* = 0.40 (*n*-hexane: ethyl acetate, 6:4); purity (HPLC): 99 %; mp: 180-182 °C; FTIR cm^-1^: 3375 (broad OH stretch), 1606 (C=O), 822 (C-Cl); ^1^H NMR (500 MHz, CDCl_3_) δ (ppm): 10.04 (s, 1H, -COOH), 8.54 (s, 1H,-CH), 7.89-7.81 (m, 2H, Aromatic -CH), 7.80-7.75 (m, 2H, Aromatic -CH), 7.45-7.36 (m,3H, Aromatic -CH), 7.53-7.47 (m, 2H, Aromatic -CH); ^13^C NMR (500MHz, CDCl_3_) δ (ppm): 171.82 (1C), 158.76 (1C), 149.03 (1C), 140.05 (1C), 139.75 (1C), 138.46 (1C), 130.75 (1C), 129.69 (2C), 128.27 (2C), 127.88 (2C), 119.64 (2C), 114.17 (1C); MS (ESI): *m/z *299.20 [m+1]^+^.

#### 3-(4-fluorophenyl)-1-phenyl-1H-pyrazole-4-carboxylic acid (10c)

10c was synthesized using Na_2_HPO_4 _(0.01 mol), potassium permanganate (0.05 mol), 9c (0.015mol) as mentioned in the general procedure to yield 10c as white crystals. Yield: 80 %; TLC R*f* = 0.58 (*n*-hexane: ethyl acetate, 6:4); purity (HPLC): 98 %; mp: 170-174^0^C; FTIR cm^-1^: 3392 (broad OH stretch, 1666.53 (C=O), 1219 (C-F);^ 1^H NMR (500 MHz, CDCl_3_) δ (ppm): δ 8.57 (s, 1H, -CH), 7.90 (dd, 2H, J=8.4, 5.4, Aromatic -CH), 7.78 (d, 2H, J=7.9, Aromatic -CH), 7.51 (d, 2H, J=7.8, Aromatic -CH), 7.39 (t, 1H, J=7.5, Aromatic -CH), 7.14 (t, 2H, J=8.6, Aromatic -CH), ^13^C NMR (500 MHz, CDCl_3_) δ (ppm): 169 (1C), 165 (1C), 146 (1C), 139.05 (1C), 133.36 (1C), 131.36 (2C), 131.30 (1C), 129.68 (2C), 127.82 (2C), 119.63 (2C), 115.12 (1C), 114.94 (1C), MS (ESI): *m/z *283.25 [m+1]^+^.

### In-vitro cytotoxicity assay

#### Cell culture

The human C6 cell line used for the bioassay was procured from the National Centre for Cell Sciences (NCCS), Pune, India. Upon arrival, early-passaged cells were cryopreserved in liquid nitrogen and then maintained as working stocks with minimal passaging to preserve the integrity of the cell line. Cells were cultured in Ham's F-12K complete media (HiMedia) supplemented with 10 % fetal bovine serum (FBS; Sigma Aldrich, Merck) and 50 mg/mL of penicillin-streptomycin (HiMedia). Cultured were maintained at 37 °C in a humidified atmosphere with 5 % CO_2_. Additional reagents used during cell culture included Dulbecco's Phosphate Buffered Saline (DPBS; Hi-media), Trypsin (Himedia), and Trypan Blue (HiMedia). 

#### Cytotoxicity assay

**Figure 7:**
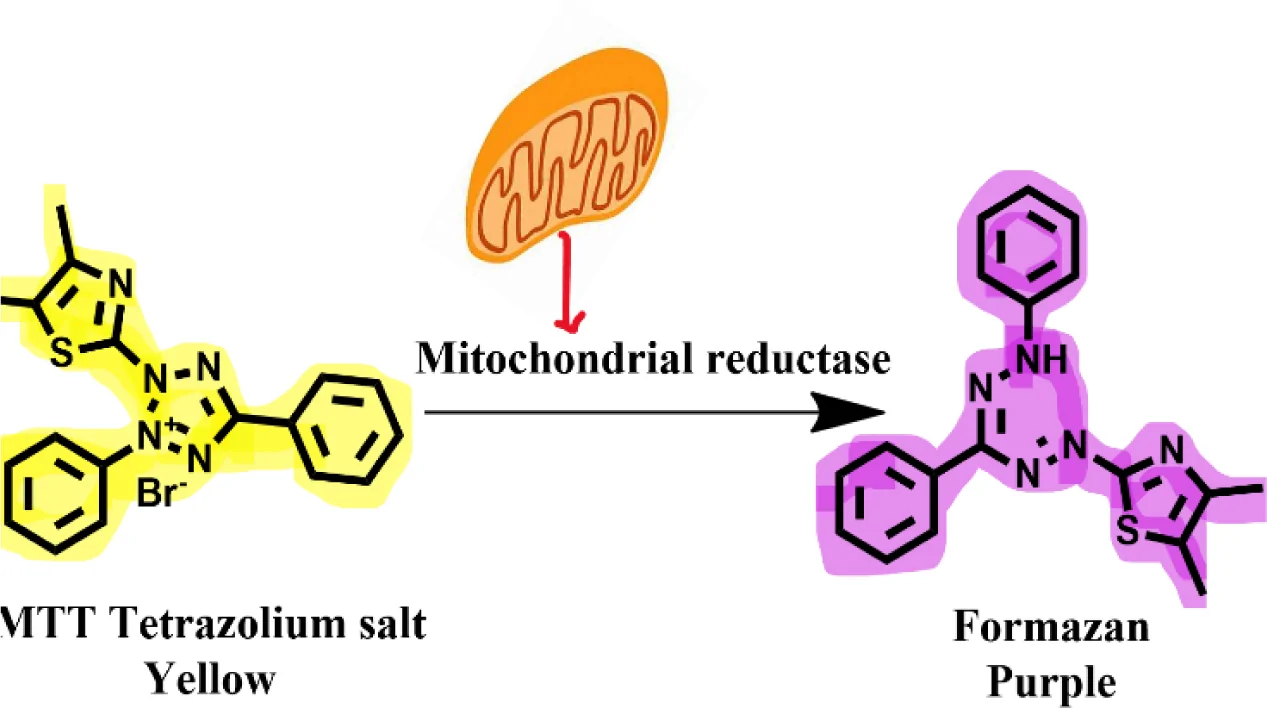
Reduction of MTT by mitochondrial reductase enzyme

**Figure 8:**
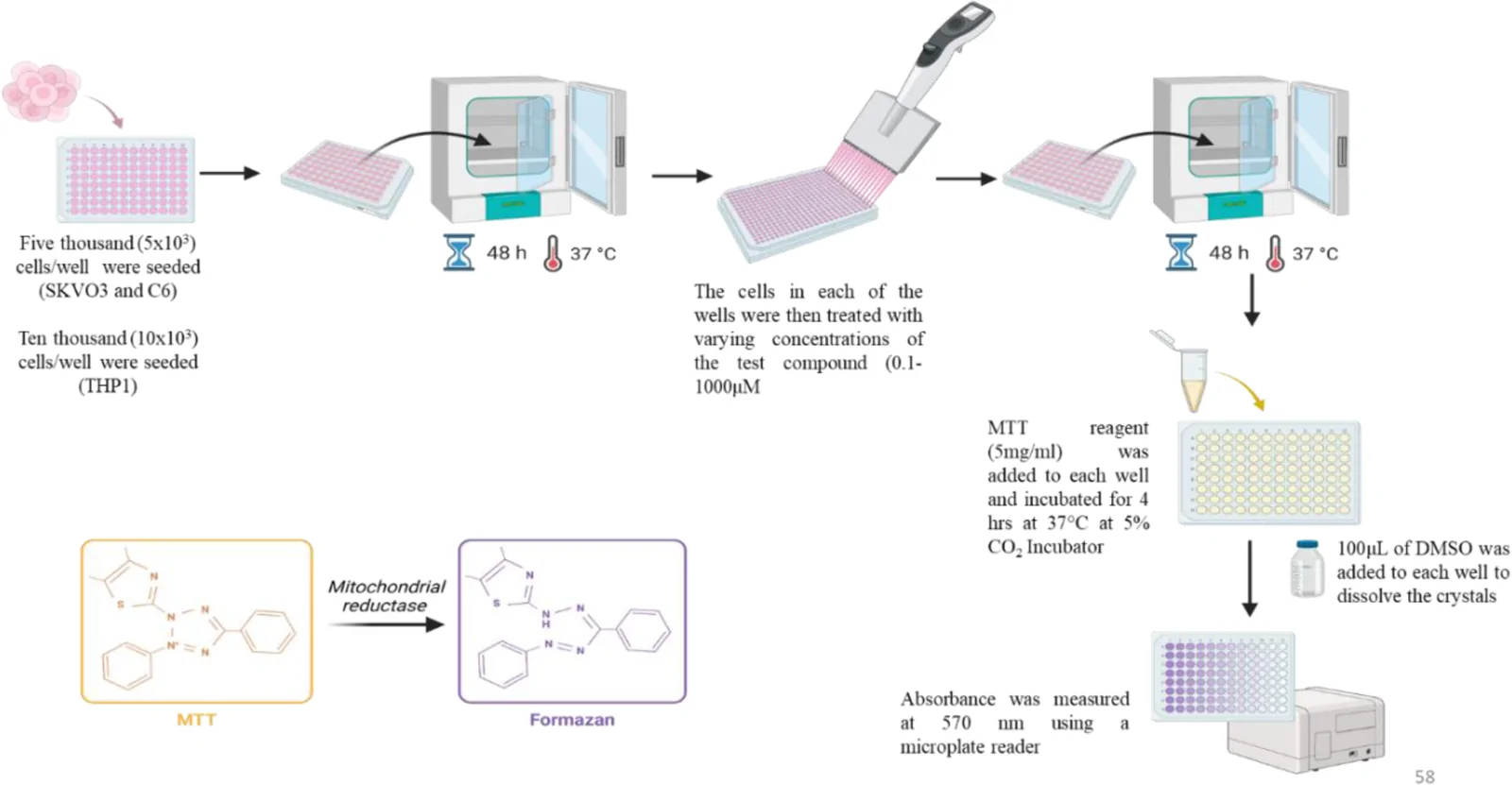
Cytotoxic study by MTT (3-(4,5-dimethylthiazol-2-yl)-2,5-diphenyltetrazolium bromide) assay

*AURK* possess oncogenic properties largely due to their central role in the regulation of mitosis, contributing to cancer cell survival and proliferation. Cytotoxicity testing of the synthesized compounds was assessed by MTT assay using three human cell lines, namely SKOV3, C6 and THP 1 cells. In the MTT assay, cellular metabolic activity is quantified as an indicator of cell viability, proliferation and cytotoxicity. In this colorimetric assay, yellow tetrazolium salt is reduced by metabolically active cells to insoluble purple formazan crystals (Figure 7(Fig. 7) and 8(Fig. 8)). The formazan crystals are then dissolved, and developed colored solution is measured in a plate reader at 570 nm. The absorbance is directly proportional to the number of living, metabolically active cells in the culture. 

#### Cytotoxicity study on ovarian cancer (SKOV3) cell line

*In-vitro* cytotoxicity was measured for compounds 2a-10f using the MTT assay in the SKOV3 cell line. Cells were seeded into 96-wells plates at a density of 5x10^3^ cells/well and incubated for 48 hours at 37 °C at 5 % CO_2 _incubator. The cells in each of the wells were then treated with varying concentrations of the test compound (0.1-1000 μM). After 48 hr incubation, 10 µl of MTT reagent (5 mg/ml in PBS) was added to each well and incubated for 4 hours at 37 °C at 5 % CO_2_ Incubator. Then supernatants were removed carefully from the wells without disturbing the formazan crystals, and 100μL of DMSO was added to each well to dissolve the crystals with gentle pipetting. Absorbance was measured at 570 nm using a microplate reader (van Meerloo et al., 2011[17]). The standard used in this assay was gemcitabine (50 µM) for SKOV3 and cell viability (%) was determined using the following formula: 

Viability % = [OD (Treated) / OD (control)] * 100

Dose-response curves were constructed, and IC_50_ values were calculated using non-linear regression analysis. All data represent the mean ± S.D. of three independent experiments. 

#### Cytotoxicity study on brain cancer (C6) cell line

The MTT-based cytotoxicity assay of the compounds 2a-10f was performed in C6 cell line. Five thousand (5x10^3^) cells/well were seeded in a 96-well plate and were incubated for 48 hours at 37 °C at 5 % CO_2 _Incubator. The cells in each of the wells were then treated with varying concentrations of the test compound (0.1-1000 μM). After 48 hr incubation, 10 µl of MTT reagent (5 mg/ml in PBS) was added to each well and incubated for 4 hrs at 37 °C at 5 % CO_2_ Incubator. Then supernatants were removed carefully from the wells without disturbing the formazan crystals and 100 μL of DMSO was added to each well to dissolve the crystals with gentle pipetting. Absorbance was measured at 570 nm using a microplate reader (van Meerloo et al., 2011[17]). The standard used in this assay was doxorubicin (0.1-1000 nM) for C6 and cell viability ( %) was determined using the following formula: 

Viability % = [OD (Treated) / OD (control)] * 100

Dose-response curves were constructed, and IC_50_ values were calculated using non-linear regression analysis. All data represent the mean ± S.D. of three independent experiments. 

#### Cytotoxicity study on leukemia (THP1) cell line

The MTT-based cytotoxicity assay of the compounds 2a-10f was performed in THP1 cell line. Ten thousand (10x10^3^) cells/well were seeded in a 96-well plate and were incubated for 48 hours at 37 °C at 5 % CO_2 _Incubator. The cells in each of the wells were then treated with varying concentrations of the test compound (0.1-1000 μM). After 48 hr incubation, 10 µl of MTT reagent (5 mg/ml in PBS) was added to each well and incubated for 4 hrs at 37 °C at 5 % CO_2_ Incubator. Then supernatants were removed carefully from the wells without disturbing the formazan crystals and 100 μL of DMSO was added to each well to dissolve the crystals with gentle pipetting. Absorbance was measured at 570 nm using a microplate reader (van Meerloo et al., 2011[17]). The standard used in this assay was paclitaxel (50 nM) for THP1 and cell viability (%) was determined using the following formula: 

Viability % = [OD (Treated) / OD (control)] * 100

Dose-response curves were constructed, and IC_50_ values were calculated using non-linear regression analysis. All data represent the mean ± S.D. of three independent experiments. 

### In-vitro AURKA and AURKB kinase inhibition study

**Figure 9:**
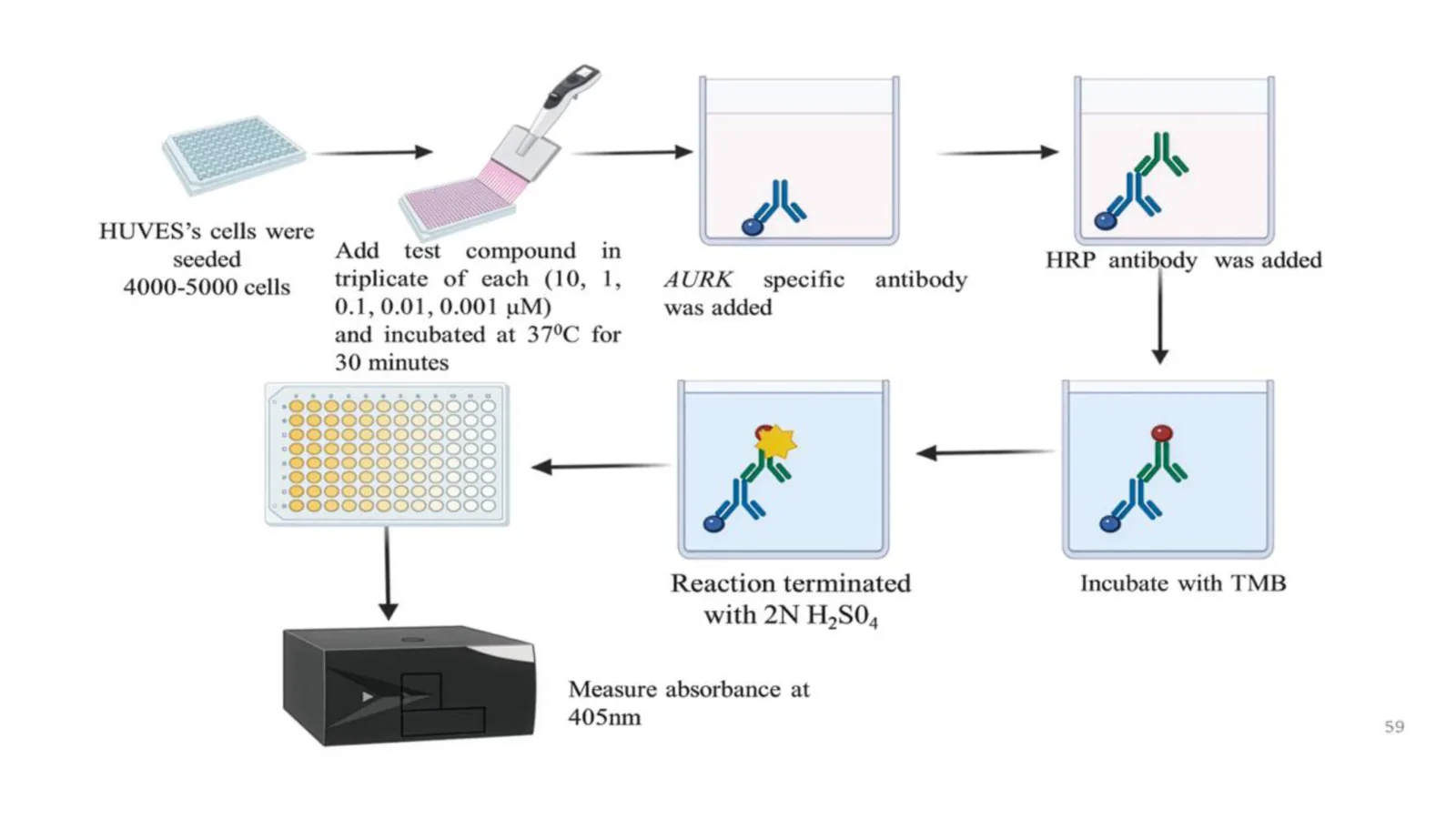
*In-vitro* *AURKA* and *AURKB* kinase inhibition study

Human umbilical vein endothelial cells (HUVEC) were seeded into a 96-well plates at a density of 4000-5000 cells per well using DMEM medium with 10 % fetal calf serum (FCS) for cell-based ELISA analysis. The cells were serum deprived for 24 hours after 12 hours of incubation. The test samples were then mixed with serum and incubated at 37 °C for 30 minutes. After removing the culture media, the cells were fixed with 4 % formaldehyde prepared in PBS and subsequently washed with PBS three time. Cells were treated with anti-*AURKA*, anti-*AURKB*, antibody 1:1000 for 1 hour. After three washes with PBS, the cells were treated with an HRP conjugated secondary antibody (1:5000) for 1 hour. Following additional rinsing, TMB substrate was added, and the reaction was stopped using 2N H_2_SO_4_. Absorbance was measured at the 405 nm. The percentage inhibition was calculated by normalizing absorbance values to the control (Figure 9(Fig. 9)) (Hardwicke et al., 2009[6]).

### Molecular docking study

The molecular docking study of 2, 4-diaminopyrimidines, pyrazole-4-carbaldehydes, and pyrazole-4- carboxylic acids was carried out using Glide module software (Schrodinger's Maestro v13.6) (Schrödinger, 2023[15]). The X-ray crystallographic structures of *AURKA *and* AURKB* were retrieved from the protein data bank, with accession Id 3UP7 (RCSB, Protein Data Bank, 2011[10]) and 4AF3 (RCSB, Protein Data Bank, 2012[11]), respectively. The protein preparation wizard of the Glide software (Schrodinger's Maestro v13.6) (Schrödinger, 2023[16]) was used for the assignment of the hydrogen bonds, additions of the hydrogens, deletion of water beyond 5 Aº from the het group. The refinement step is crucial, as it is involved in optimizing hydrogen bonded groups and energy minimization by using default force field OPLS4. The Glide grid file was generated using receptor grid generation panel by picking up the co-crystallized ligand located at the active site of the proteins. Glide automatically calculates the default grid centre and dimensions based on the ligand's spatial location and overall size. The receptor grid had X, Y, and Z coordinates with 10.00, 11.00, 10.00 for *AURKA *and 16.00, 15.00, 16.00 for *AURKB*, respectively. These coordinates indicate the enclosing box in which ligand molecules interact with the protein. The structures of the synthesized compounds were used in MOL format. The compounds were prepared using the LigPrep module in Schrodinger's Maestro software (Schrödinger, 2023[14]). Minimization of the compounds was carried out using the OPLS4 force field module. The docking of the compounds was carried out using Glide's ligand docking module (Schrödinger, 2023[13]) and the standard precision (SP) was used for visualization of docking score. The synthesized compounds were evaluated based on their G-score, interactions at the active site with key amino acids and interactions of the co-crystallized ligand with *AURKA *and *AURKB*, respectively.

### In-silico physiochemical and ADME predication studies

*In-silico* physiochemical and ADME attributes of the synthesized compounds were predicted using QikProp, Maestro, Schrodinger.

## Declaration

### Acknowledgments 

The authors thank DST-FIST for providing equipment support and Schrodinger licence support for the execution of this project (DST/FST/College-054/2017).

### Conflict of interest 

The authors declare no conflict of interest.

### Artificial Intelligence (AI) statement

The authors declare no AI tools were used in the design, conduct, analysis, and interpretation of the research reported in the manuscript. 

## Supplementary Material

Supplementary data

## Figures and Tables

**Table 1:**
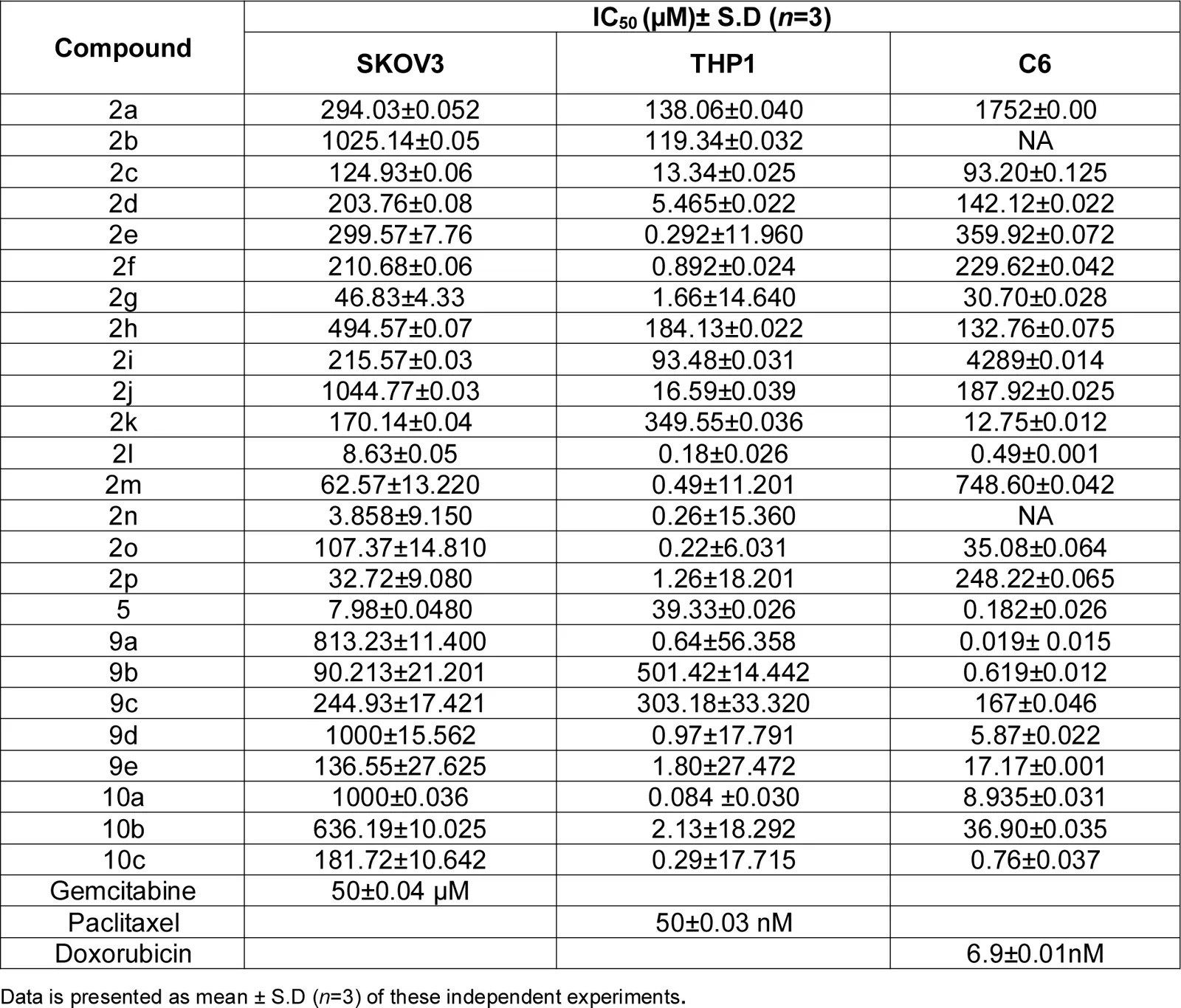
Cytotoxicity activity of synthesized compounds in SKOV3, THP1, and C6 cell lines

**Table 2:**
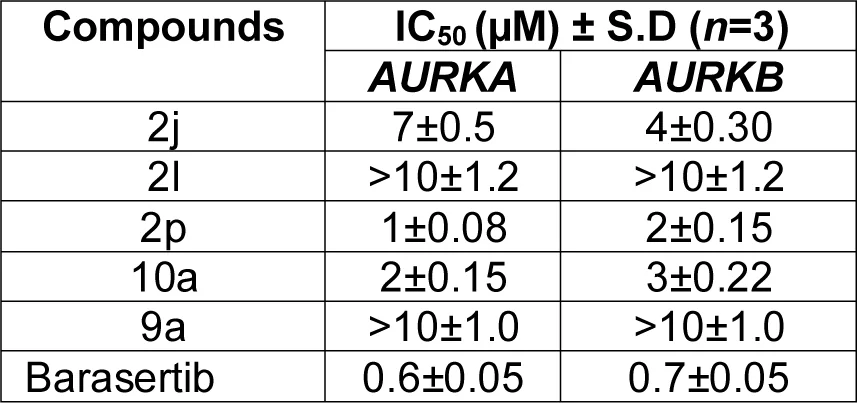
*In-vitro*2 *AURKA* and *AURKB* inhibition assay

**Table 3:**
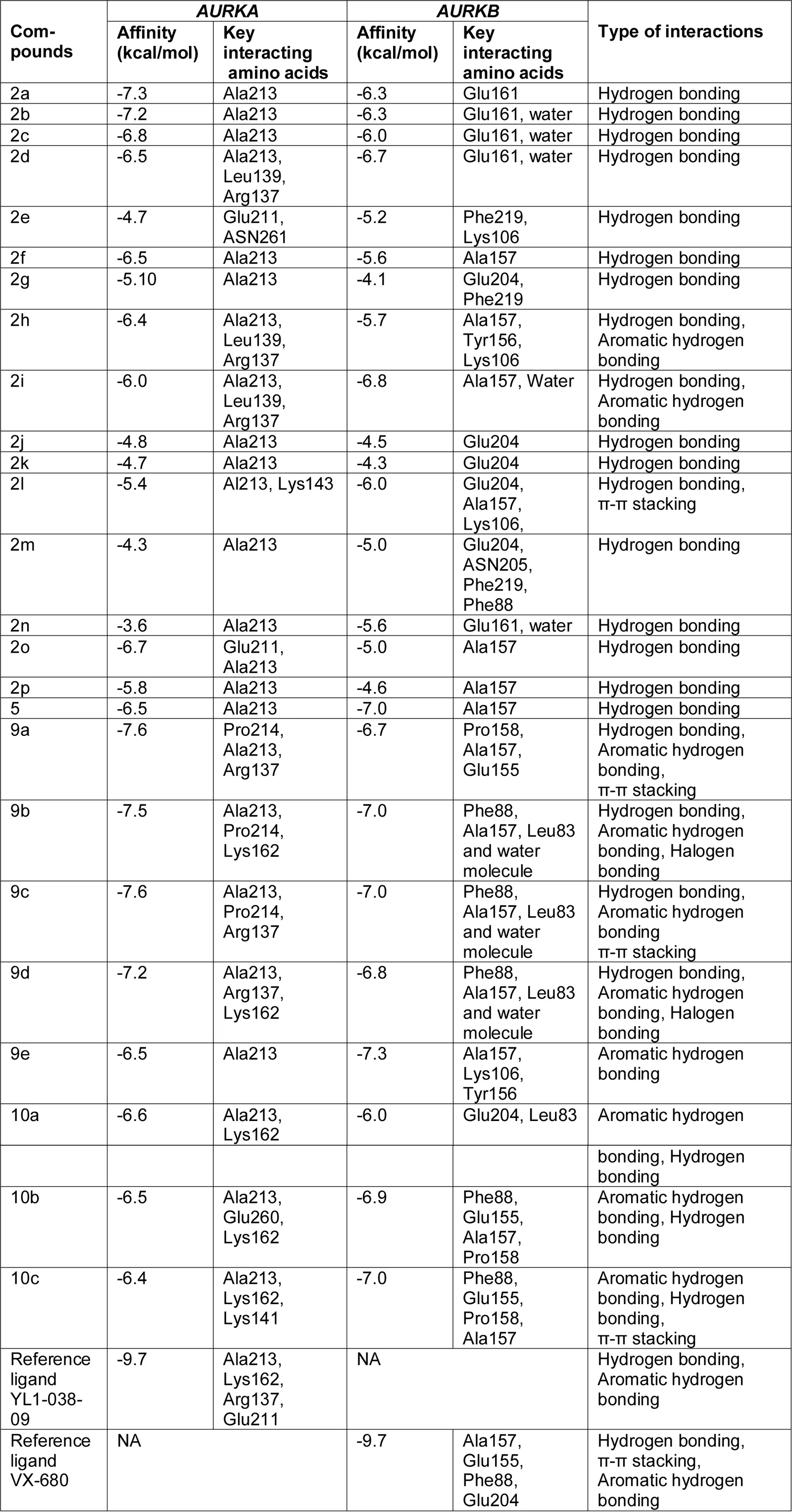
Docking binding affinity (energy) and interactions at the active site of *AURKA* (3UP7) and *AURKB *(4AF3) kinase.

**Table 4:**
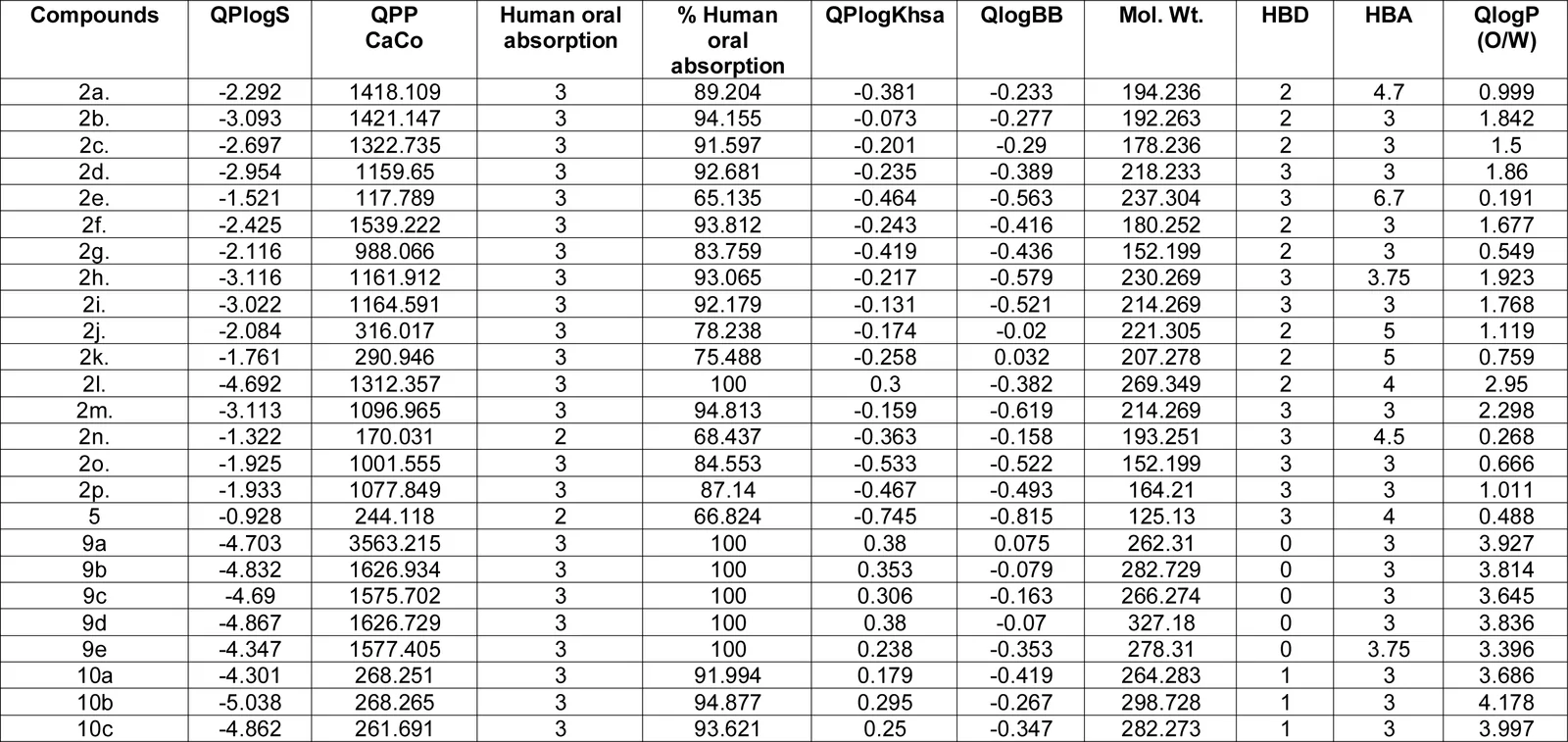
ADME prediction study of synthesized compounds

**Table 5:**
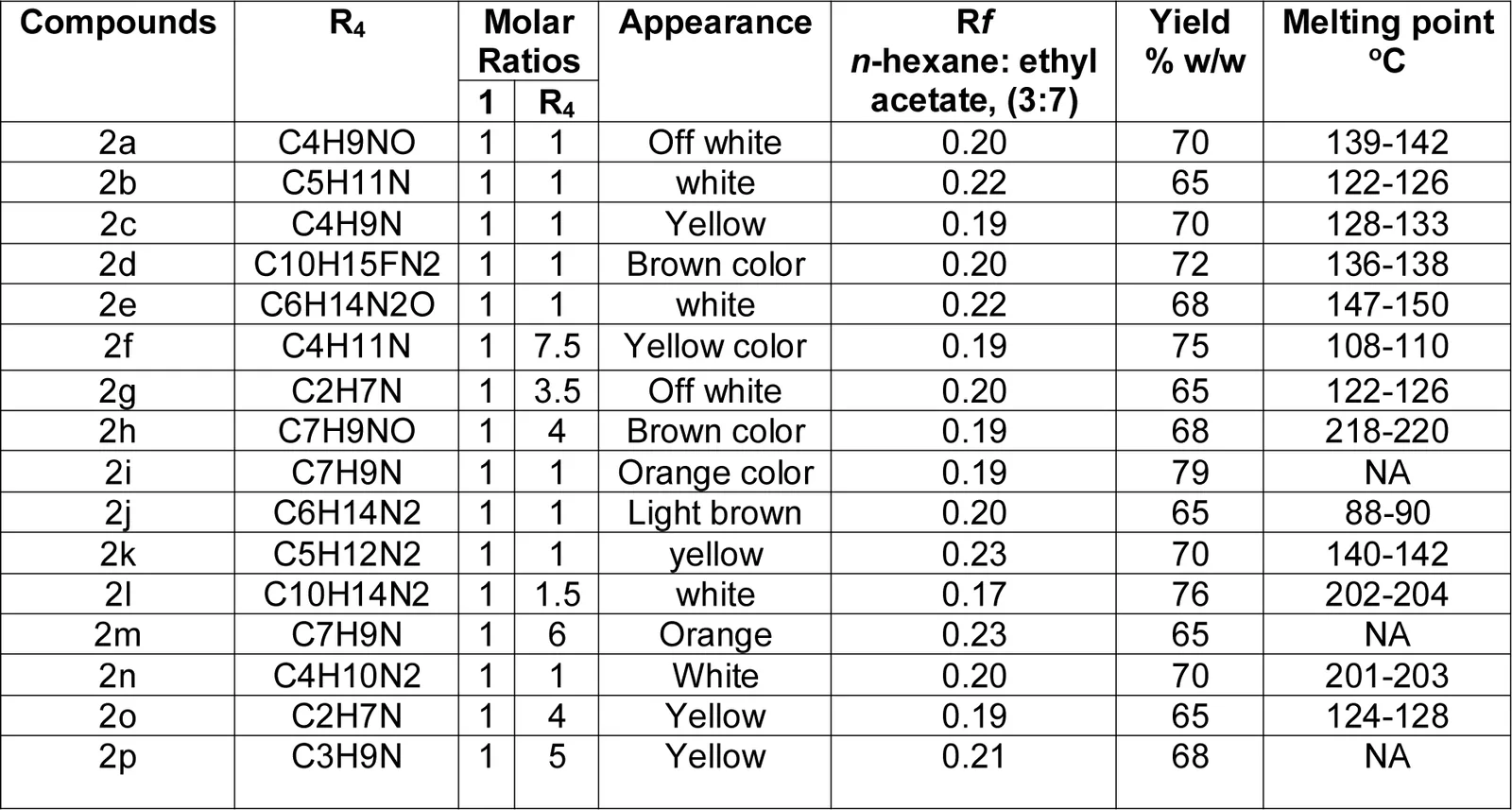
Physiochemical data of 2,4-diaminopyrimidines

**Table 6:**
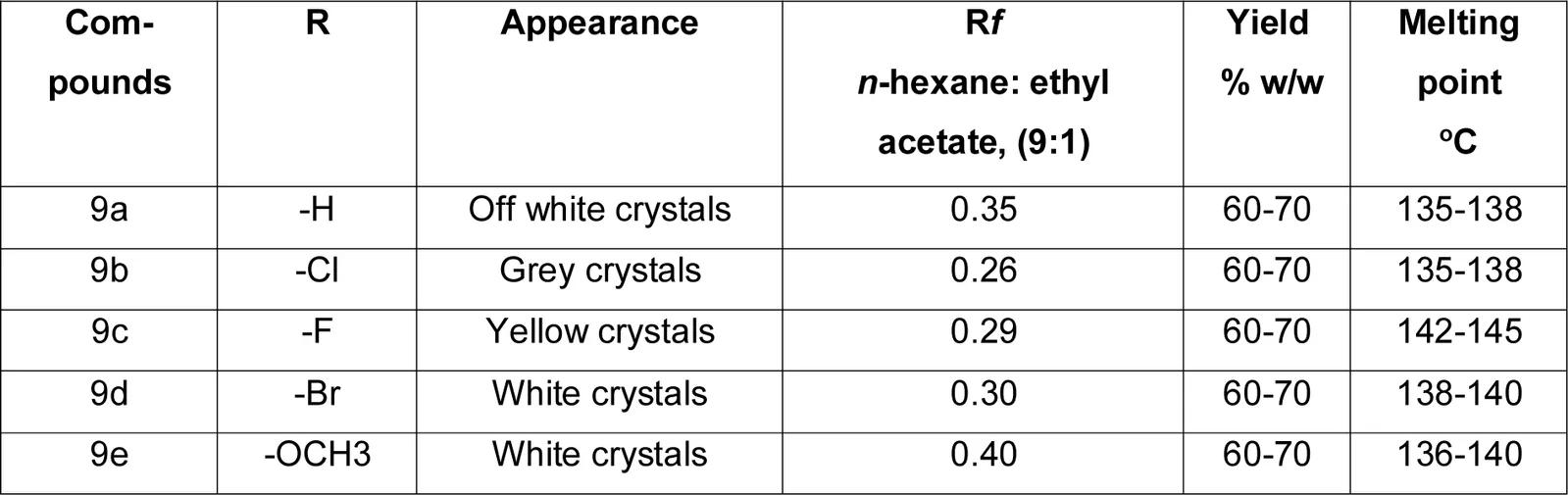
Physiochemical data of synthesized pyrazole-4-carbaldehydes

**Table 7:**
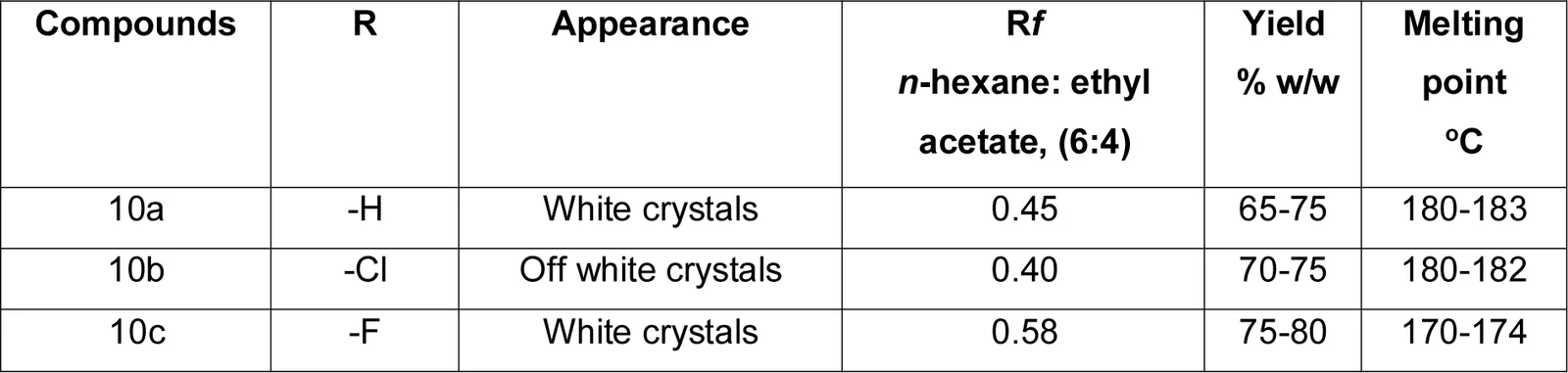
Physiochemical data of synthesized pyrazole-4-carboxylic acid

**Figure 1:**
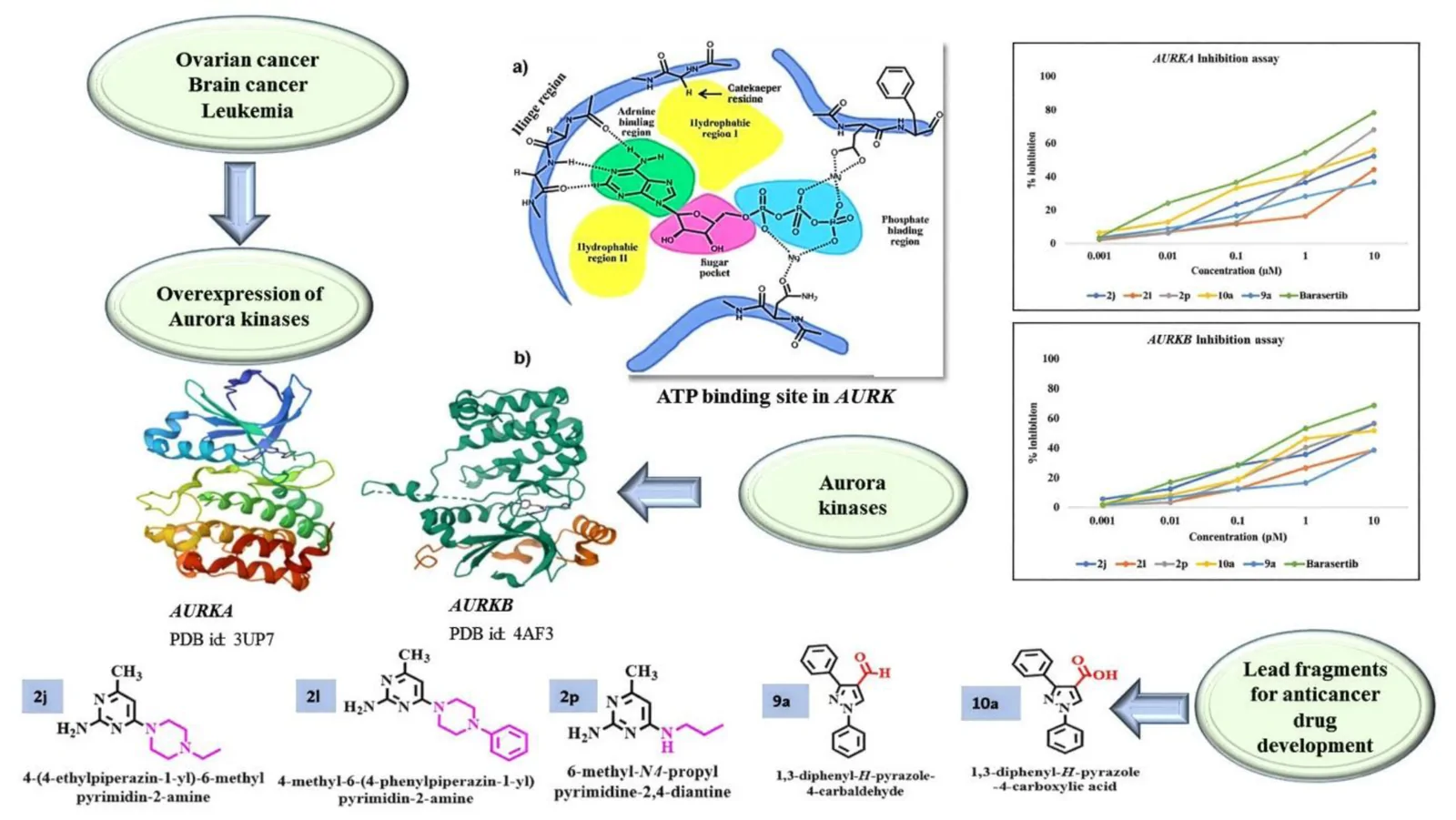
Graphical abstract

## References

[1] Abdel-Wahab BF, Khidre RE, Farahat AA (2011). Pyrazole-3(4)-carbaldehyde: synthesis, reactions and biological activity. Arkivoc.

[2] Bayramoğlu D, Kurtay G, Güllü M (2020). Ultrasound-assisted rapid synthesis of 2-aminopyrimidine and barbituric acid derivatives. Synth Commun.

[3] Bolanos-Garcia VM (2005). Aurora kinases. Int J Biochem Cell Biol.

[4] Bratenko MK, Chornous VA, Vovk MV (2001). 4-Functionally substituted 3-heterylpyrazoles: III. 3-aryl(heteryl)pyrazole-4-carboxylic acids and their derivatives. Russ J Org Chem.

[5] Du R, Huang C, Liu K, Li X, Dong Z (2021). Targeting AURKA in Cancer: molecular mechanisms and opportunities for Cancer therapy. Mol Cancer.

[6] Hardwicke MA, Oleykowski CA, Plant R, Wang J, Liao Q, Moss K (2009). GSK 1070916, a potent Aurora B/C kinase inhibitor with broad antitumor activity in tissue culture cells and human tumor xenograft models. Mol Cancer Ther.

[7] Kovacs AH, Zhao D, Hou J (2023). Aurora B. Inhibitors as Cancer Therapeutics. Molecules.

[8] Quartuccio SM, Schindler K (2015). Functions of Aurora kinase C in meiosis and cancer. Front Cell Dev Biol.

[9] Qureshi F, Nawaz M, Hisaindee S, Almofty SA, Ansari MA, Jamal QMS (2022). Microwave assisted synthesis of 2-amino-4-chloro-pyrimidine derivatives: anticancer and computational study on potential inhibitory action against COVID-19. Arab J Chem.

[10] RCSB Protein Data Bank (2011). 3UP7, Aurora A in complex with YL1-038-09. https://www.rcsb.org/structure/3UP7.

[11] RCSB Protein Data Bank (2012). 4AF3, Human Aurora B kinase in complex with INCENP and VX-680. https://www.rcsb.org/structure/4AF3.

[12] Sankhe K, Prabhu A, Khan T (2021). Design strategies, SAR, and mechanistic insight of Aurora kinase inhibitors in cancer. Chem Biol Drug Des.

[13] Schrödinger Release 2023-4 (2023). Glide.

[14] Schrödinger Release 2023-4 (2023). LigPrep.

[15] Schrödinger Release 2023-4 (2023). Maestro.

[16] Schrödinger Release 2023-4 (2023). Protein Preparation Wizard; Epik; Impact; Prime.

[17] van Meerloo J, Kaspers GJL, Cloos J, Cree IA (2011). Cell sensitivity assays: the MTT assay. Cancer Cell Culture: Methods and Protocols.

[18] World Health Organization (2023). Cancer. https://www.who.int/news-room/fact-sheets/detail/cancer.

