# Supplementary material for: Synthesis, molecular docking and biological evaluation of 2,4-diaminopyrimidines and pyrazoles as aurora kinase inhibitors
Source: EXCLI J. 2026 Aug 9;25:1316–45. doi: 10.17179/excli2026-9347 (PMC13595871; doi:10.17179/excli2026-9347)

EXCLI Journal 2026;25:1316-1345 – ISSN 1611-2156
Received: February 17, 2026, accepted: June 01, 2026, published: August 09, 2026

Supplementary data to:

Original article:

SYNTHESIS, MOLECULAR DOCKING AND BIOLOGICAL EVALUATION OF 2,4-DIAMINOPYRIMIDINES AND PYRAZOLES AS AURORA KINASE INHIBITORS

Kaksha Sankhe, Mrunal Jadhav, Arati Prabhu, Tabassum Khan*

Department of Pharmaceutical Chemistry, SVKM's Dr. Bhanuben Nanavati College of Pharmacy, University of Mumbai, Mumbai, Maharashtra, India

* **Corresponding author:** Dr. Tabassum Khan, Professor, Department of Pharmaceutical Chemistry & Quality Assurance, SVKM's Dr. Bhanuben Nanavati College of Pharmacy, Vile Parle West, Mumbai- 400 056, Maharashtra, India. Tel.: 9198924775606, E-mail: tabassum.khan@bncp.ac.in

https://dx.doi.org/10.17179/excli2026-9347

This is an Open Access article distributed under the terms of the Creative Commons Attribution License (https://creativecommons.org/licenses/by/4.0/).

Supplementary Table 1: Raw data

Raw absorbance data obtained from the MTT cytotoxicity assay performed on the SKOV3 ovarian cancer cell line. These data were used to calculate the percentage cell viability and IC_{50} values reported in Table 1 of the main manuscript.

1. Cytotoxicity study on SKOV3 cell line

Com-pounds	Concentration µM	Absorbance readings (*n = 3*)			Corrected OD	Average % Viability	SD
		T1	T2	T3			
**2a**	Control	0.381	0.161	0.19	0.255	100.000	0.085
	1000	0.169	0.159	0.31	0.170	66.907	0.084
	100	0.33	0.323	0.331	0.286	112.209	0.004
	10	0.329	0.398	0.344	0.315	123.601	0.036
	1	0.347	0.288	0.244	0.251	98.462	0.052
	0.1	0.374	0.4	0.304	0.317	124.517	0.050
	Blank	0.041	0.044	0.042			
**2b**	Control	0.427	0.444	0.462	0.386	100.000	0.088
	1000	0.089	0.081	0.132	0.047	12.168	0.027
	100	0.362	0.29	0.376	0.289	74.822	0.046
	10	0.393	0.399	0.413	0.348	90.097	0.010
	1	0.484	0.375	0.439	0.379	98.123	0.055

EXCLI Journal 2026;25:1316-1345 – ISSN 1611-2156
Received: February 17, 2026, accepted: June 01, 2026, published: August 09, 2026

	0.1	0.521	0.592	0.442	0.465	120.302	0.075
	Blank	0.059	0.056	0.046			
**2c**	Control	0.666	0.539	0.387	0.386	100.000	0.088
	1000	0.146	0.206	0.086	0.092	23.905	0.060
	100	0.364	0.41	0.297	0.303	78.533	0.057
	10	0.423	0.417	0.306	0.328	85.005	0.066
	1	0.312	0.423	0.293	0.289	74.822	0.070
	0.1	0.351	0.429	0.311	0.310	80.259	0.060
	Blank	0.059	0.056	0.046			
**2d**	Control	0.549	0.532	0.571	0.413	100.000	0.086
	1000	0.106	0.102	0.093	-0.014	-3.391	0.007
	100	0.503	0.563	0.652	0.458	110.999	0.075
	10	0.589	0.577	0.758	0.527	127.629	0.101
	1	0.592	0.63	1.011	0.630	152.573	0.232
	0.1	0.538	0.538	0.545	0.426	103.169	0.004
	Blank	0.123	0.106	0.114			
**2e**	Control	0.687	0.75	0.731	0.525	100.000	0.083
	1000	0.533	0.575	0.607	0.458	87.232	0.037
	100	0.57	0.59	0.594	0.471	89.707	0.013
	10	0.644	0.696	0.55	0.517	98.335	0.074
	1	0.658	0.623	0.659	0.533	101.507	0.021
	0.1	0.61	0.697	0.566	0.511	97.256	0.067
	Blank	0.11	0.104	0.126			
**2f**	Control	0.293	0.483	0.636	0.413	100.000	0.086
	1000	0.113	0.108	0.103	-0.006	-1.534	0.005
	100	0.451	0.409	0.625	0.381	92.190	0.115
	10	0.479	0.613	0.555	0.435	105.267	0.067
	1	0.568	0.594	0.584	0.468	113.259	0.013
	0.1	0.706	0.569	0.565	0.499	120.848	0.080
	Blank	0.123	0.106	0.114			
**2g**	Control	0.474	0.365	0.39	0.284	100.000	0.000
	1000	0.3585	0.36	0.357	0.275	96.848	0.529
	100	0.3745	0.397	0.352	0.291	102.486	7.929
	10	0.3885	0.402	0.375	0.305	107.420	4.757
	1	0.412	0.42	0.404	0.328	115.701	2.819
	0.1	0.392	0.376	0.408	0.308	108.653	5.638
**2h**	Control	0.424	0.424	0.34	0.386	100.000	0.088
	1000	0.287	0.234	0.288	0.216	55.922	0.031
	100	0.303	0.371	0.368	0.294	76.030	0.038
	10	0.32	0.463	0.301	0.308	79.655	0.089
	1	0.332	0.462	0.339	0.324	83.883	0.073
	0.1	0.561	0.362	0.304	0.355	91.996	0.135
	Blank	0.059	0.056	0.046			

EXCLI Journal 2026;25:1316-1345 – ISSN 1611-2156
Received: February 17, 2026, accepted: June 01, 2026, published: August 09, 2026

**2i**	Control	0.396	0.272	0.23	0.255	100.000	0.085
	1000	0.065	0.059	0.109	0.035	13.879	0.027
	100	0.321	0.326	0.287	0.269	105.663	0.021
	10	0.289	0.322	0.324	0.269	105.794	0.020
	1	0.314	0.281	0.273	0.247	97.021	0.022
	0.1	0.436	0.4	0.437	0.382	150.049	0.021
	Blank	0.041	0.044	0.042			
**2j**	Control	0.427	0.264	0.376	0.255	100.000	0.085
	1000	0.106	0.14	0.107	0.075	29.591	0.019
	100	0.234	0.255	0.272	0.211	83.011	0.019
	10	0.247	0.325	0.355	0.267	104.746	0.056
	1	0.338	0.283	0.333	0.276	108.282	0.030
	0.1	0.483	0.32	0.284	0.320	125.696	0.106
	Blank	0.041	0.044	0.042			
**2k**	Control	0.286	0.255	0.325	0.255	100.000	0.085
	1000	0.095	0.121	0.099	0.063	24.615	0.014
	100	0.256	0.274	0.263	0.222	87.201	0.009
	10	0.371	0.24	0.318	0.267	105.008	0.066
	1	0.295	0.268	0.34	0.259	101.604	0.036
	0.1	0.322	0.323	0.365	0.294	115.614	0.025
	Blank	0.041	0.044	0.042			
**2l**	Control	0.625	0.514	0.545	0.413	100.000	0.086
	1000	0.114	0.124	0.116	0.004	0.888	0.005
	100	0.123	0.09	0.359	0.076	18.486	0.147
	10	0.288	0.3	0.288	0.178	43.027	0.007
	1	0.544	0.6	0.636	0.479	116.004	0.046
	0.1	0.556	0.541	0.528	0.427	103.491	0.014
	Blank	0.123	0.106	0.114			
**2m**	Control	0.474	0.365	0.39	0.284	100.000	0.000
	1000	0.183	0.205	0.244	0.099	44.753	10.886
	100	0.25	0.286	0.309	0.166	69.773	10.479
	10	0.265	0.284	0.317	0.181	72.240	9.272
	1	0.289	0.348	0.37	0.205	88.802	14.760
	0.1	0.325	0.427	0.427	0.241	109.005	20.752
**2n**	Control	0.474	0.365	0.39	0.284	100.000	0.000
	1000	0.523	0.537	0.501	0.439	101.998	4.239
	100	0.529	0.543	0.669	0.445	116.013	18.011
	10	0.538	0.521	0.542	0.454	105.113	2.605
	1	0.594	0.725	0.676	0.510	135.790	15.461
	0.1	0.543	0.554	0.588	0.459	111.653	5.480

EXCLI Journal 2026;25:1316-1345 – ISSN 1611-2156
Received: February 17, 2026, accepted: June 01, 2026, published: August 09, 2026

**2o**	Control	0.474	0.365	0.39	0.284	100.000	0.000
	1000	0.379	0.466	0.513	0.295	86.193	15.881
	100	0.439	0.534	0.488	0.355	94.212	11.097
	10	0.446	0.53	0.569	0.362	100.753	14.682
	1	0.434	0.542	0.574	0.350	101.142	17.135
	0.1	0.488	0.619	0.556	0.404	109.940	15.303
**2p**	Control	0.474	0.365	0.39	0.284	100.000	0.000
	1000	0.45	0.48	0.492	0.366	91.176	5.053
	100	0.432	0.437	0.532	0.348	89.541	13.162
	10	0.482	0.554	0.499	0.398	99.974	8.791
	1	0.513	0.461	0.512	0.429	96.159	6.946
	0.1	0.502	0.447	0.545	0.418	96.782	11.474
**9a**	Control	0.474	0.365	0.39	0.284	100.000	0.000
	1000	0.397	0.34	0.375	0.313	101.135	10.130
	100	0.488	0.456	0.485	0.404	138.371	6.228
	10	0.465	0.463	0.428	0.381	129.796	7.333
	1	0.538	0.477	0.589	0.454	158.927	19.760
	0.1	0.533	0.484	0.457	0.449	143.657	13.576
**9b**	Control	0.474	0.365	0.39	0.284	100.000	0.000
	1000	0.277	0.347	0.335	0.200	80.442	12.409
	100	0.331	0.385	0.415	0.254	99.448	14.111
	10	0.378	0.521	0.507	0.301	129.834	26.132
	1	0.478	0.556	0.545	0.401	148.950	13.995
	0.1	0.358	0.599	0.445	0.281	129.392	40.456
**9c**	Control	0.474	0.365	0.39	0.390	100.000	0.000
	1000	0.281	0.212	0.196	0.197	44.280	7.038
	100	0.326	0.284	0.302	0.242	68.188	5.287
	10	0.443	0.458	0.389	0.359	108.263	21.807
	1	0.51	0.48	0.453	0.426	123.555	16.037
	0.1	0.508	0.581	0.433	0.424	133.175	37.855
**9d**	Control	0.474	0.365	0.39	0.284	100.000	0.000
	1000	0.456	0.308	0.426	0.379	105.967	25.936
	100	0.382	0.398	0.439	0.305	109.171	9.746
	10	0.444	0.5	0.477	0.367	131.492	9.331
	1	0.466	0.442	0.39	0.389	117.901	12.878
	0.1	0.354	0.333	0.446	0.277	99.669	19.924
**9e**	Control	0.474	0.365	0.39	0.284	100.000	0.000
	1000	0.56	0.484	0.6	0.483	156.133	19.533
	100	0.619	0.46	0.572	0.542	156.906	27.078
	10	0.573	0.628	0.594	0.496	172.818	9.201
	1	0.612	0.456	0.779	0.535	178.564	53.546
	0.1	0.623	0.504	0.673	0.546	173.370	28.779

**10a**	Control	0.261	0.28	0.3	0.233	100.000	0.033
	1000	0.067	0.066	0.056	0.001	0.572	0.006
	100	0.207	0.279	0.261	0.187	80.401	0.037
	10	0.381	0.329	0.34	0.288	123.748	0.027
	1	0.294	0.296	0.263	0.223	95.565	0.019
	0.1	0.232	0.418	0.305	0.257	110.157	0.094
	Blank	0.085	0.051	0.049			
**10b**	Control	0.474	0.365	0.39	0.284	100.000	0.000
	1000	0.397	0.352	0.442	0.320	130.199	18.309
	100	0.508	0.522	0.531	0.431	180.380	4.716
	10	0.479	0.509	0.567	0.402	179.566	18.202
	1	0.534	0.537	0.513	0.457	183.499	5.321
	0.1	0.498	0.511	0.495	0.421	172.649	3.460
**10c**	Control	0.474	0.365	0.39	0.284	100.000	0.000
	1000	0.249	0.256	0.254	0.165	63.606	1.354
	100	0.358	0.313	0.289	0.274	88.773	13.158
	10	0.281	0.335	0.314	0.197	85.017	10.225
	1	0.292	0.322	0.344	0.208	88.523	9.805
	0.1	0.251	0.343	0.33	0.167	84.265	18.702

EXCLI Journal 2026;25:1316-1345 – ISSN 1611-2156
Received: February 17, 2026, accepted: June 01, 2026, published: August 09, 2026

2. Cytotoxicity study on THP1 cell line

Raw absorbance data obtained from the MTT cytotoxicity assay performed on the THP1 leukemia cancer cell line. These data were used to calculate the percentage cell viability and IC_{50} values reported in Table 1 of the main manuscript.

Compounds	Concentrations µM	Absorbance readings (*n = 3*)			Corrected OD	Average % Viability	SD
		T1	T2	T3			
2a	Control	0.742	0.75	0.701	0.241	100	0.120
	1000	0.613	0.618	0.614	0.094	39.142	0.003
	100	0.779	0.705	0.751	0.224	93.084	0.037
	10	0.752	0.766	0.75	0.235	97.649	0.009
	1	0.781	0.747	0.789	0.252	104.426	0.022
	0.1	0.741	0.751	0.838	0.256	106.224	0.053
	Blank	0.508	0.544	0.51			
2b	Control	0.742	0.75	0.701	0.077	100.000	0.043
	1000	0.551	0.549	0.528	0.022	28.448	0.013
	100	0.587	0.54	0.666	0.077	99.569	0.064
	10	0.596	0.647	0.627	0.103	132.759	0.026
	1	0.591	0.619	0.668	0.105	136.207	0.039
	0.1	0.604	0.617	0.622	0.094	121.121	0.009
	Blank	0.508	0.544	0.51			
2c	Control	0.742	0.75	0.701	0.108	100.000	0.076
	1000	0.649	0.679	0.67	0.007	6.180	0.015
	100	0.729	0.76	0.748	0.086	80.037	0.016
	10	0.741	0.745	0.733	0.080	74.475	0.006
	1	0.781	0.745	0.73	0.093	85.909	0.026
	0.1	0.75	0.731	0.727	0.077	71.075	0.012
	Blank	0.64	0.644	0.694			
2d	Control	0.742	0.75	0.701	0.112	100.000	0.029
	1000	0.661	0.634	0.643	0.048	42.687	0.014
	100	0.682	0.647	0.617	0.050	45.075	0.033
	10	0.724	0.671	0.669	0.090	80.299	0.031
	1	0.752	0.667	0.664	0.096	85.970	0.050
	0.1	0.755	0.722	0.733	0.138	123.881	0.017
	Blank	0.566	0.574	0.655			
2e	Control	0.742	0.75	0.701	0.234	100.000	0.032
	1000	0.73	0.676	0.72	0.188	80.380	0.029
	100	0.737	0.719	0.742	0.212	90.641	0.012
	10	0.777	0.721	0.739	0.225	96.200	0.029
	1	0.795	0.78	0.739	0.251	107.173	0.029
	0.1	0.789	0.736	0.784	0.249	106.461	0.029
	Blank	0.508	0.544	0.51			

EXCLI Journal 2026;25:1316-1345 – ISSN 1611-2156
Received: February 17, 2026, accepted: June 01, 2026, published: August 09, 2026

**2f**	Control	0.742	0.75	0.701	0.108	100.000	0.076
	1000	0.706	0.67	0.68	0.026	24.104	0.019
	100	0.742	0.73	0.744	0.079	73.548	0.008
	10	0.753	0.707	0.716	0.066	61.187	0.024
	1	0.726	0.729	0.712	0.063	58.405	0.009
	0.1	0.737	0.72	0.725	0.068	63.041	0.009
	Blank	0.64	0.644	0.694			
**2g**	Control	0.304	0.363	0.385	0.095	100.000	0.000
	1000	0.283	0.317	0.327	0.033	62.105	24.279
	100	0.284	0.31	0.312	0.034	54.737	16.443
	10	0.3	0.337	0.306	0.050	67.719	20.903
	1	0.331	0.344	0.338	0.081	92.281	6.849
	0.1	0.359	0.363	0.368	0.109	119.298	4.747
**2h**	Control	0.742	0.75	0.701	0.106	100.000	0.025
	1000	0.59	0.581	0.554	0.054	51.097	0.019
	100	0.65	0.66	0.632	0.127	119.122	0.014
	10	0.632	0.672	0.642	0.128	120.376	0.021
	1	0.638	0.629	0.697	0.134	126.019	0.037
	0.1	0.684	0.646	0.66	0.143	134.169	0.019
	Blank	0.508	0.544	0.51			
**2i**	Control	0.742	0.75	0.701	0.077	100.000	0.043
	1000	0.635	0.511	0.545	0.043	55.603	0.064
	100	0.599	0.547	0.58	0.055	70.690	0.026
	10	0.581	0.593	0.607	0.073	94.397	0.013
	1	0.65	0.591	0.572	0.084	108.190	0.041
	0.1	0.59	0.593	0.593	0.071	92.241	0.002
	Blank	0.508	0.544	0.51			
**2j**	Control	0.742	0.75	0.701	0.241	100.000	0.120
	1000	0.561	0.615	0.657	0.090	37.483	0.048
	100	0.63	0.673	0.662	0.134	55.740	0.022
	10	0.72	0.7	0.695	0.184	76.487	0.013
	1	0.732	0.739	0.714	0.208	86.169	0.013
	0.1	0.787	0.806	0.766	0.266	110.235	0.020
	Blank	0.508	0.544	0.51			
**2k**	Control	0.742	0.75	0.701	0.241	100.000	0.120
	1000	0.75	0.662	0.685	0.178	73.997	0.046
	100	0.804	0.725	0.771	0.246	102.075	0.040
	10	0.808	0.799	0.805	0.283	117.566	0.005
	1	0.827	0.83	0.832	0.309	128.216	0.003
	0.1	0.844	0.848	0.834	0.321	133.333	0.007
	Blank	0.508	0.544	0.51			

**2l**	Control	0.742	0.75	0.701	0.108	100.000	0.076
	1000	0.652	0.665	0.67	0.003	2.781	0.009
	100	0.68	0.647	0.661	0.003	3.090	0.017
	10	0.703	0.68	0.672	0.026	23.795	0.016
	1	0.711	0.731	0.693	0.052	48.517	0.019
	0.1	0.733	0.714	0.762	0.077	71.384	0.024
	Blank	0.64	0.644	0.694			
**2m**	Control	0.363	0.331	0.333	0.095	100.000	0.000
	1000	0.325	0.292	0.287	0.075	54.035	21.735
	100	0.314	0.294	0.301	0.064	55.789	10.683
	10	0.315	0.298	0.309	0.065	60.351	9.075
	1	0.304	0.301	0.31	0.054	57.895	4.824
	0.1	0.366	0.35	0.35	0.116	110.877	9.724
**2n**	Control	0.374	0.375	0.36	0.095	100.000	0.000
	1000	0.3	0.292	0.266	0.050	38.136	18.831
	100	0.3	0.302	0.273	0.050	44.138	17.158
	10	0.29	0.301	0.273	0.040	40.254	14.944
	1	0.289	0.31	0.287	0.039	48.023	13.497
	0.1	0.371	0.353	0.349	0.121	114.054	12.414
**2o**	Control	0.344	0.363	0.379	0.095	100.000	0.000
	1000	0.3	0.303	0.321	0.050	61.441	12.032
	100	0.31	0.31	0.31	0.060	63.559	0.000
	10	0.3	0.305	0.313	0.050	59.322	6.946
	1	0.309	0.313	0.31	0.059	64.266	2.205
	0.1	0.35	0.363	0.347	0.100	109.463	9.009
**2p**	Control	0.341	0.381	0.381	0.095	100.000	0.000
	1000	0.264	0.281	0.29	0.014	30.014	13.987
	100	0.271	0.303	0.312	0.021	48.023	22.827
	10	0.325	0.303	0.3	0.075	62.853	14.460
	1	0.32	0.305	0.3	0.070	61.794	11.026
	0.1	0.311	0.358	0.358	0.061	97.811	28.745
**9a**	Control	0.366	0.329	0.338	0.095	100.000	0.000
	1000	0.284	0.27	0.287	0.034	31.930	31.930
	100	0.298	0.276	0.28	0.048	36.491	36.491
	10	0.296	0.299	0.304	0.046	52.281	52.281
	1	0.332	0.292	0.295	0.082	59.298	59.298
	0.1	0.337	0.339	0.364	0.087	101.754	101.754
**9b**	Control	0.342	0.312	0.316	0.095	100.000	0.000
	1000	0.53	0.491	0.49	0.280	315.768	28.395
	100	0.321	0.306	0.307	0.071	76.349	10.440
	10	0.305	0.296	0.3	0.055	62.656	5.613
	1	0.302	0.302	0.317	0.052	70.954	10.780
	0.1	0.374	0.352	0.349	0.124	134.855	16.992

EXCLI Journal 2026;25:1316-1345 – ISSN 1611-2156
Received: February 17, 2026, accepted: June 01, 2026, published: August 09, 2026

**9c**	Control	0.342	0.361	0.325	0.095	100.000	0.000
	1000	0.677	0.747	0.682	0.427	475.789	41.107
	100	0.318	0.372	0.378	0.068	111.579	34.785
	10	0.282	0.346	0.368	0.032	86.316	47.028
	1	0.306	0.362	0.372	0.056	101.754	37.444
	0.1	0.37	0.38	0.385	0.120	135.088	8.040
**9d**	Control	0.361	0.371	0.287	0.095	100.000	0.000
	1000	0.33	0.309	0.292	0.080	75.104	23.695
	100	0.33	0.321	0.308	0.080	86.722	13.768
	10	0.335	0.301	0.301	0.085	77.593	24.436
	1	0.349	0.312	0.335	0.099	102.075	23.255
	0.1	0.365	0.361	0.367	0.115	142.324	3.803
**9e**	Control	0.312	0.326	0.346	0.095	100.000	0.000
	1000	0.298	0.278	0.293	0.048	49.378	12.956
	100	0.31	0.287	0.305	0.060	63.071	15.058
	10	0.294	0.287	0.318	0.044	61.826	20.239
	1	0.319	0.291	0.354	0.069	88.797	39.292
	0.1	0.359	0.294	0.367	0.109	112.033	49.839
**10a**	Control	0.742	0.75	0.701	0.108	100.000	0.076
	1000	0.707	0.732	0.668	0.043	39.864	0.032
	100	0.693	0.697	0.665	0.026	23.795	0.017
	10	0.736	0.73	0.714	0.067	62.423	0.011
	1	0.746	0.73	0.71	0.069	64.277	0.018
	0.1	0.766	0.728	0.718	0.078	72.311	0.025
	Blank	0.64	0.644	0.694			
**10b**	Control	0.361	0.36	0.383	0.095	100.000	0.000
	1000	0.318	0.307	0.32	0.068	68.856	7.415
	100	0.364	0.317	0.332	0.114	92.867	25.431
	10	0.354	0.326	0.321	0.104	88.630	18.841
	1	0.369	0.329	0.33	0.119	98.164	24.164
	0.1	0.371	0.381	0.352	0.121	125.000	15.605
**10c**	Control	0.377	0.377	0.364	0.095	100.000	0.000
	1000	0.377	0.38	0.372	0.127	112.020	3.584
	100	0.359	0.321	0.314	0.109	72.118	21.471
	10	0.368	0.341	0.307	0.118	78.621	27.104
	1	0.37	0.312	0.323	0.120	75.369	27.316
	0.1	0.373	0.379	0.393	0.123	116.749	9.100

EXCLI Journal 2026;25:1316-1345 – ISSN 1611-2156
Received: February 17, 2026, accepted: June 01, 2026, published: August 09, 2026

3. Cytotoxicity study on C6 cell line

Raw absorbance data obtained from the MTT cytotoxicity assay performed on the C6 glioblastoma cancer cell line. These data were used to calculate the percentage cell viability and IC_{50} values reported in Table 1 of the main manuscript.

Compounds	Concentration µM	Absorbance readings (*n = 3)*			Corrected OD	Average % Viability	SD
		T1	T2	T3			
**2a**	Blank	0.112	0.112	0.135			
	Control	0.715	0.695	0.693	0.581	100.000	0.012
	1000	0.137	0.136	0.124	0.013	2.179	0.007
	100	0.189	0.205	0.215	0.083	14.335	0.013
	10	0.233	0.233	0.231	0.113	19.381	0.001
	1	0.199	0.185	0.199	0.075	12.844	0.008
	0.1	0.161	0.167	0.156	0.042	7.167	0.006
**2b**	Blank	0.112	0.112	0.135			
	Control	0.715	0.695	0.693	0.581	100.000	0.012
	1000	0.195	0.186	0.2	0.074	12.729	0.007
	100	0.227	0.225	0.218	0.104	17.833	0.005
	10	0.49	0.406	0.389	0.309	53.096	0.054
	1	0.195	0.253	0.251	0.113	19.495	0.033
	0.1	0.207	0.174	0.2	0.074	12.729	0.017
**2c**	Blank	0.112	0.112	0.135			
	Control	0.715	0.695	0.693	0.581	100.000	0.012
	1000	0.173	0.176	0.167	0.052	9.002	0.005
	100	0.182	0.185	0.213	0.074	12.672	0.017
	10	0.32	0.244	1.189	0.465	79.931	0.525
	1	0.308	0.243	0.284	0.159	27.294	0.033
	0.1	0.352	0.441	0.371	0.268	46.158	0.047
**2d**	Blank	0.112	0.112	0.135			
	Control	0.715	0.695	0.693	0.581	100.000	0.012
	1000	0.182	0.202	0.205	0.077	13.188	0.013
	100	0.376	0.372	0.379	0.256	44.037	0.004
	10	0.42	0.379	0.346	0.262	45.069	0.037
	1	0.309	0.318	0.303	0.190	32.741	0.008
	0.1	0.229	0.249	0.317	0.145	25.000	0.046
**2e**	Blank	0.112	0.112	0.135			
	Control	0.715	0.695	0.693	0.581	100.000	0.012
	1000	0.208	0.232	0.282	0.121	20.814	0.038
	100	0.2	0.282	0.375	0.166	28.555	0.088
	10	0.354	0.349	0.432	0.259	44.495	0.047
	1	0.224	0.312	0.402	0.193	33.200	0.089
	0.1	0.235	0.385	0.204	0.155	26.663	0.097

EXCLI Journal 2026;25:1316-1345 – ISSN 1611-2156
Received: February 17, 2026, accepted: June 01, 2026, published: August 09, 2026

**2f**	Blank	0.112	0.112	0.135			
	Control	0.715	0.695	0.693	0.581	100.000	0.012
	1000	0.337	0.316	0.266	0.187	32.110	0.036
	100	0.443	0.384	0.459	0.309	53.154	0.040
	10	0.494	0.468	0.479	0.361	62.041	0.013
	1	0.455	0.651	0.546	0.431	74.140	0.098
	0.1	0.498	0.389	0.484	0.337	58.028	0.059
**2g**	Blank	0.112	0.112	0.135			
	Control	0.715	0.695	0.693	0.581	100.000	0.012
	1000	0.184	0.195	0.199	0.073	12.557	0.008
	100	0.172	0.171	0.175	0.053	9.117	0.002
	10	0.197	0.227	0.277	0.114	19.610	0.040
	1	0.255	0.269	0.224	0.130	22.305	0.023
	0.1	0.183	0.187	0.25	0.087	14.966	0.038
**2h**	Blank	0.112	0.112	0.135			
	Control	0.715	0.695	0.693	0.581	100.000	0.012
	1000	0.195	0.197	0.215	0.083	14.220	0.011
	100	0.303	0.282	0.367	0.198	34.002	0.044
	10	0.451	0.505	0.498	0.365	62.787	0.029
	1	0.484	0.498	0.326	0.316	54.415	0.096
	0.1	0.336	0.289	0.68	0.315	54.243	0.213
**2i**	Blank	0.112	0.112	0.135			
	Control	0.715	0.695	0.693	0.581	100.000	0.012
	1000	0.295	0.287	0.242	0.155	26.663	0.029
	100	0.187	0.19	0.192	0.070	12.041	0.003
	10	0.26	0.267	0.282	0.150	25.803	0.011
	1	0.237	0.221	0.268	0.122	21.044	0.024
	0.1	0.207	0.221	0.21	0.093	15.998	0.007
**2j**	Blank	0.112	0.112	0.135			
	Control	0.715	0.695	0.693	0.581	100.000	0.012
	1000	0.129	0.124	0.127	0.007	1.204	0.003
	100	0.277	0.254	0.231	0.134	23.108	0.023
	10	0.228	0.256	0.282	0.136	23.337	0.027
	1	0.227	0.296	0.308	0.157	27.064	0.044
	0.1	0.248	0.257	0.324	0.157	26.950	0.042
**2k**	Blank	0.112	0.112	0.135			
	Control	0.715	0.695	0.693	0.581	100.000	0.012
	1000	0.128	0.129	0.119	0.006	0.975	0.006
	100	0.144	0.149	0.154	0.029	5.046	0.005
	10	0.205	0.222	0.2	0.089	15.367	0.012
	1	0.259	0.261	0.229	0.130	22.362	0.018
	0.1	0.264	0.276	0.298	0.160	27.466	0.017

**2l**	Blank	0.112	0.112	0.135			
	Control	0.715	0.695	0.693	0.581	100.000	0.012
	1000	0.133	0.131	0.122	0.009	1.548	0.006
	100	0.133	0.136	0.135	0.015	2.580	0.002
	10	0.123	0.129	0.132	0.008	1.433	0.005
	1	0.169	0.176	0.179	0.055	9.461	0.005
	0.1	0.23	0.265	0.227	0.121	20.814	0.021
**2m**	Blank	0.112	0.112	0.135			
	Control	0.715	0.695	0.693	0.581	100.000	0.012
	1000	0.127	0.31	0.106	0.061	10.550	0.112
	100	0.296	0.376	0.352	0.222	38.131	0.041
	10	0.444	0.444	0.454	0.328	56.365	0.006
	1	0.434	0.437	0.454	0.322	55.390	0.011
	0.1	0.237	0.303	0.346	0.176	30.218	0.055
**2n**	Blank	0.112	0.112	0.135			
	Control	0.715	0.695	0.693	0.581	100.000	0.012
	1000	0.189	0.194	0.191	0.072	12.328	0.003
	100	0.232	0.241	0.245	0.120	20.585	0.007
	10	0.196	0.289	0.24	0.122	20.986	0.047
	1	0.203	0.254	0.295	0.131	22.534	0.046
	0.1	0.211	0.236	0.255	0.114	19.667	0.022
**2o**	Blank	0.112	0.112	0.135			
	Control	0.715	0.695	0.693	0.581	100.000	0.012
	1000	0.184	0.194	0.183	0.067	11.583	0.006
	100	0.265	0.186	0.181	0.091	15.654	0.047
	10	0.619	0.388	0.398	0.349	59.977	0.131
	1	0.572	0.389	0.365	0.322	55.447	0.113
	0.1	0.559	0.493	0.519	0.404	69.495	0.033
**2p**	Blank	0.112	0.112	0.135			
	Untreated	0.715	0.695	0.693	0.581	100.000	0.012
	1000	0.205	0.185	0.183	0.071	12.271	0.012
	100	0.544	0.243	0.24	0.223	38.303	0.175
	10	0.342	0.313	0.249	0.182	31.250	0.048
	1	0.348	0.328	0.256	0.191	32.856	0.048
	0.1	0.322	0.281	0.244	0.163	27.982	0.039
**9a**	Blank	0.112	0.112	0.135			
	Control	0.715	0.695	0.693	0.581	100.000	0.012
	1000	0.128	0.127	0.141	0.012	2.122	0.008
	100	0.126	0.123	0.133	0.008	1.319	0.005
	10	0.157	0.139	0.155	0.031	5.275	0.010
	1	0.167	0.158	0.153	0.040	6.823	0.007
	0.1	0.323	0.314	0.42	0.233	40.023	0.059

EXCLI Journal 2026;25:1316-1345 – ISSN 1611-2156
Received: February 17, 2026, accepted: June 01, 2026, published: August 09, 2026

**9b**	Blank	0.112	0.112	0.135			
	Control	0.715	0.695	0.693	0.581	100.000	0.012
	1000	0.116	0.205	0.139	0.034	5.791	0.046
	100	0.123	0.121	0.128	0.004	0.745	0.004
	10	0.132	0.15	0.143	0.022	3.784	0.009
	1	0.137	0.165	0.153	0.032	5.505	0.014
	0.1	0.166	0.166	0.169	0.047	8.142	0.002
**9C**	Blank	0.112	0.112	0.135			
	Control	0.715	0.695	0.693	0.581	100.000	0.012
	1000	0.405	0.245	0.317	0.203	34.862	0.080
	100	0.37	0.383	0.43	0.275	47.248	0.032
	10	0.4	0.298	0.347	0.229	39.335	0.051
	1	0.402	0.414	0.405	0.287	49.427	0.006
	0.1	0.393	0.276	0.366	0.225	38.761	0.061
**9d**	Blank	0.112	0.112	0.135			
	Control	0.715	0.695	0.693	0.581	100.000	0.012
	1000	0.122	0.118	0.129	0.003	0.573	0.006
	100	0.174	0.158	0.283	0.085	14.679	0.068
	10	0.139	0.16	0.121	0.020	3.498	0.020
	1	0.151	0.153	0.146	0.030	5.218	0.004
	0.1	0.14	0.14	0.133	0.018	3.096	0.004
**9e**	Blank	0.112	0.112	0.135			
	Control	0.715	0.695	0.693	0.581	100.000	0.012
	1000	0.121	0.127	0.125	0.005	0.803	0.003
	100	0.155	0.145	0.158	0.033	5.677	0.007
	10	0.156	0.136	0.151	0.028	4.817	0.010
	1	0.223	0.203	0.186	0.084	14.507	0.019
	0.1	0.167	0.152	0.146	0.035	6.078	0.011
**10a**	Blank	0.112	0.112	0.135			
	Control	0.715	0.695	0.693	0.581	100.000	0.012
	1000	0.348	0.367	0.378	0.245	42.087	0.015
	100	0.38	0.357	0.352	0.243	41.858	0.015
	10	0.384	0.378	0.381	0.261	44.954	0.003
	1	0.409	0.52	0.403	0.324	55.791	0.066
	0.1	0.388	0.352	0.464	0.282	48.452	0.057
**10b**	Blank	0.112	0.112	0.135			
	Control	0.715	0.695	0.693	0.581	100.000	0.012
	1000	0.36	0.352	0.274	0.209	35.952	0.048
	100	0.273	0.377	0.297	0.196	33.716	0.054
	10	0.348	0.402	0.384	0.258	44.438	0.027
	1	0.392	0.402	0.41	0.282	48.452	0.009
	0.1	0.357	0.361	0.306	0.222	38.131	0.031

**10C**	Blank	0.112	0.112	0.135			
	Control	0.715	0.695	0.693	0.581	100.000	0.012
	1000	0.388	0.368	0.363	0.253	43.578	0.013
	100	0.39	0.247	0.286	0.188	32.339	0.074
	10	0.386	0.319	0.343	0.230	39.507	0.034
	1	0.409	0.352	0.325	0.242	41.686	0.043
	0.1	0.381	0.377	0.391	0.263	45.298	0.007

EXCLI Journal 2026;25:1316-1345 – ISSN 1611-2156
Received: February 17, 2026, accepted: June 01, 2026, published: August 09, 2026

Supplementary Table 2: Raw data

Raw percentage inhibition data obtained from the *in-vitro AURKA* and *AURKB* inhibition assay. These raw data were used to calculate mean percentage inhibition, standard deviation (S.D), and IC_{50} values reported in Table 2 (*in-vitro AURKA* and *AURKB* inhibition assay) of the main manuscript.

1. Target inhibition study on Aurora Kinase A

Compounds	Concentration µM	% Inhibition (*n = 3*)			Mean Average % Inhibition	S.D
		T1	T2	T3		
**2j**	10	51.4	52.8	52.9	52.367	0.685
	1	35.7	36.8	37.3	36.600	0.668
	0.1	22.9	23.7	24.3	23.633	0.573
	0.01	6	6.3	6.5	6.267	0.205
	0.001	2	2.2	2.25	2.150	0.108
**2l**	10	43.2	44.6	44.9	44.233	0.741
	1	15.8	16.6	16.7	16.367	0.403
	0.1	11.2	11.7	12	11.633	0.330
	0.01	6.3	6.5	7	6.600	0.294
	0.001	2.3	2.5	2.6	2.467	0.125
**2p**	10	66.9	68.9	68.6	68.133	0.881
	1	38.5	39.8	40.2	39.500	0.726
	0.1	12.2	12.7	13	12.633	0.330
	0.01	6.4	6.7	6.9	6.667	0.205
	0.001	3.5	3.8	3.74	3.680	0.130
**10a**	10	54.2	56	56.7	55.633	1.053
	1	41.5	42.5	42.5	42.167	0.471
	0.1	32.4	33.3	34.1	33.267	0.694
	0.01	12.3	12.9	13.3	12.833	0.411
	0.001	6.2	6.5	6.8	6.500	0.245
**9a**	10	35.5	36.8	37.6	36.633	0.865
	1	27.8	28.5	29	28.433	0.492
	0.1	16	16.6	17.3	16.633	0.531
	0.01	8.6	9	9.2	8.933	0.249
	0.001	3.3	3.5	3.7	3.500	0.163
**Standard Barasertib**	10	77.2	78.8	79.3	78.433	0.896
	1	53.4	54.9	54.5	54.267	0.634
	0.1	35.8	36.9	37.3	36.667	0.634
	0.01	23.4	24.3	24.7	24.133	0.544
	0.001	3.4	3.7	3.9	3.667	0.205

EXCLI Journal 2026;25:1316-1345 – ISSN 1611-2156
Received: February 17, 2026, accepted: June 01, 2026, published: August 09, 2026

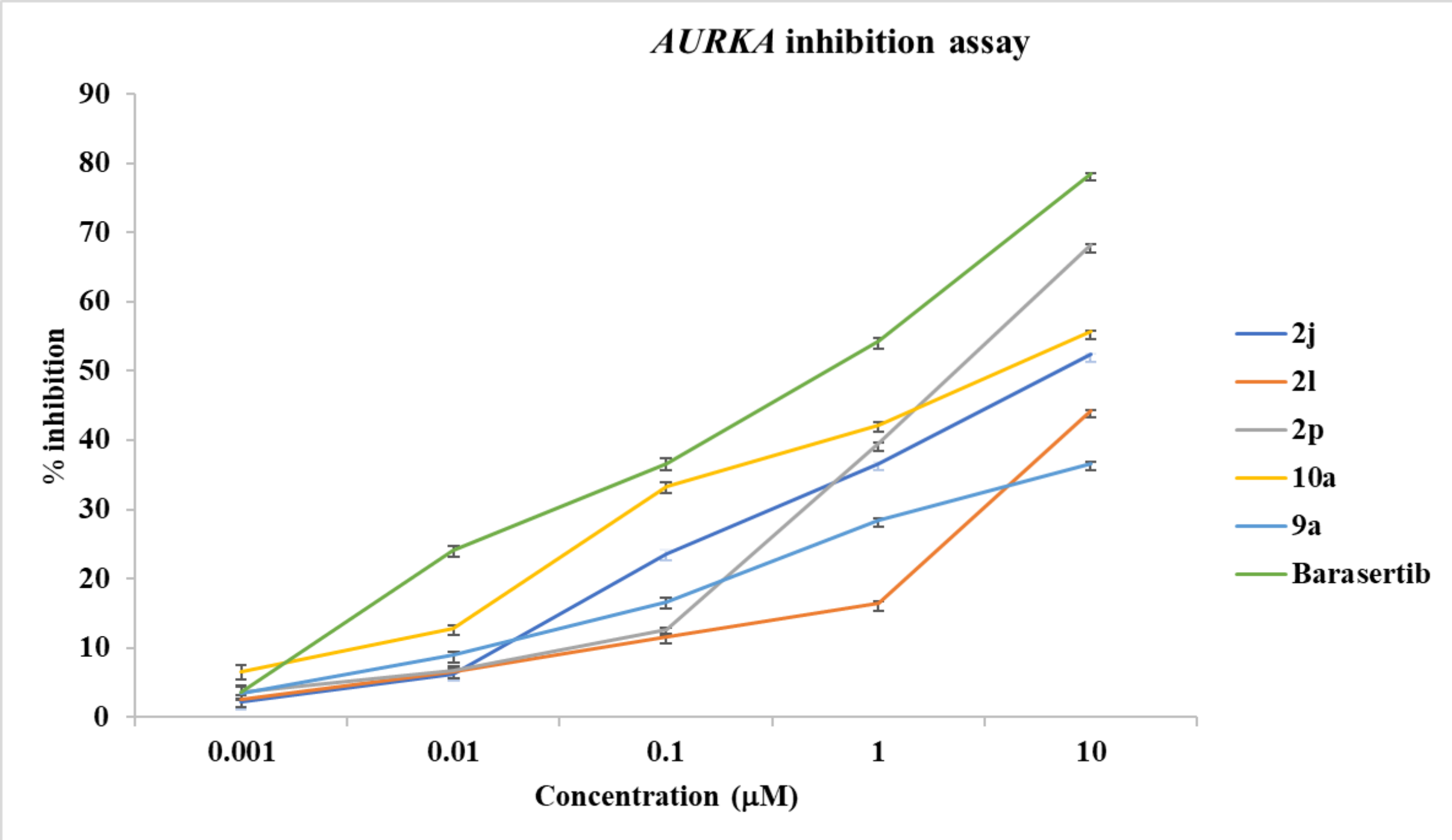

2. Target inhibition study on Aurora Kinase B

Compounds	Concentration µM	% Inhibition (n = 3)			Mean Average % Inhibition	S.D
		T1	T2	T3		
**2j**	10	55.2	56.8	57.1	56.367	0.834
	1	34.8	35.9	36.3	35.667	0.634
	0.1	27.6	28.9	29.2	28.567	0.694
	0.01	11.9	12.5	12.9	12.433	0.411
	0.001	5.2	5.5	5.7	5.467	0.205
**2l**	10	37.8	38.9	39.2	38.633	0.602
	1	25.9	26.8	27.2	26.633	0.544
	0.1	12.1	12.7	13	12.600	0.374
	0.01	3.4	3.7	3.8	3.633	0.170
	0.001	1.3	1.5	1.6	1.467	0.125
**2p**	10	55.2	56.9	57.1	56.400	0.852
	1	39.3	40.8	40.7	40.267	0.685
	0.1	17.9	18.6	19.1	18.533	0.492
	0.01	3	3.3	3.5	3.267	0.205
	0.001	1.6	1.7	1.9	1.733	0.125

**10a**	10	50.7	52.3	51.9	51.633	0.680
	1	45.5	46.9	46.28	46.227	0.573
	0.1	17.9	18.7	18.9	18.500	0.432
	0.01	7.7	8.2	8.3	8.067	0.262
	0.001	3	3.2	3.4	3.200	0.163
**9a**	10	37.5	38.9	38.8	38.400	0.638
	1	15.9	16.7	17.3	16.633	0.573
	0.1	12.1	12.7	13	12.600	0.374
	0.01	6.3	6.6	6.9	6.600	0.245
	0.001	1.3	1.5	1.6	1.467	0.125
**Standard Barasertib**	10	67.2	68.9	69.7	68.6	1.042
	1	52.1	53.8	53.6	53.166	0.758
	0.1	27.6	28.8	29.1	28.5	0.648
	0.01	16.2	17.1	17.2	16.833	0.449
	0.001	0.9	1.1	1.2	1.066	0.124

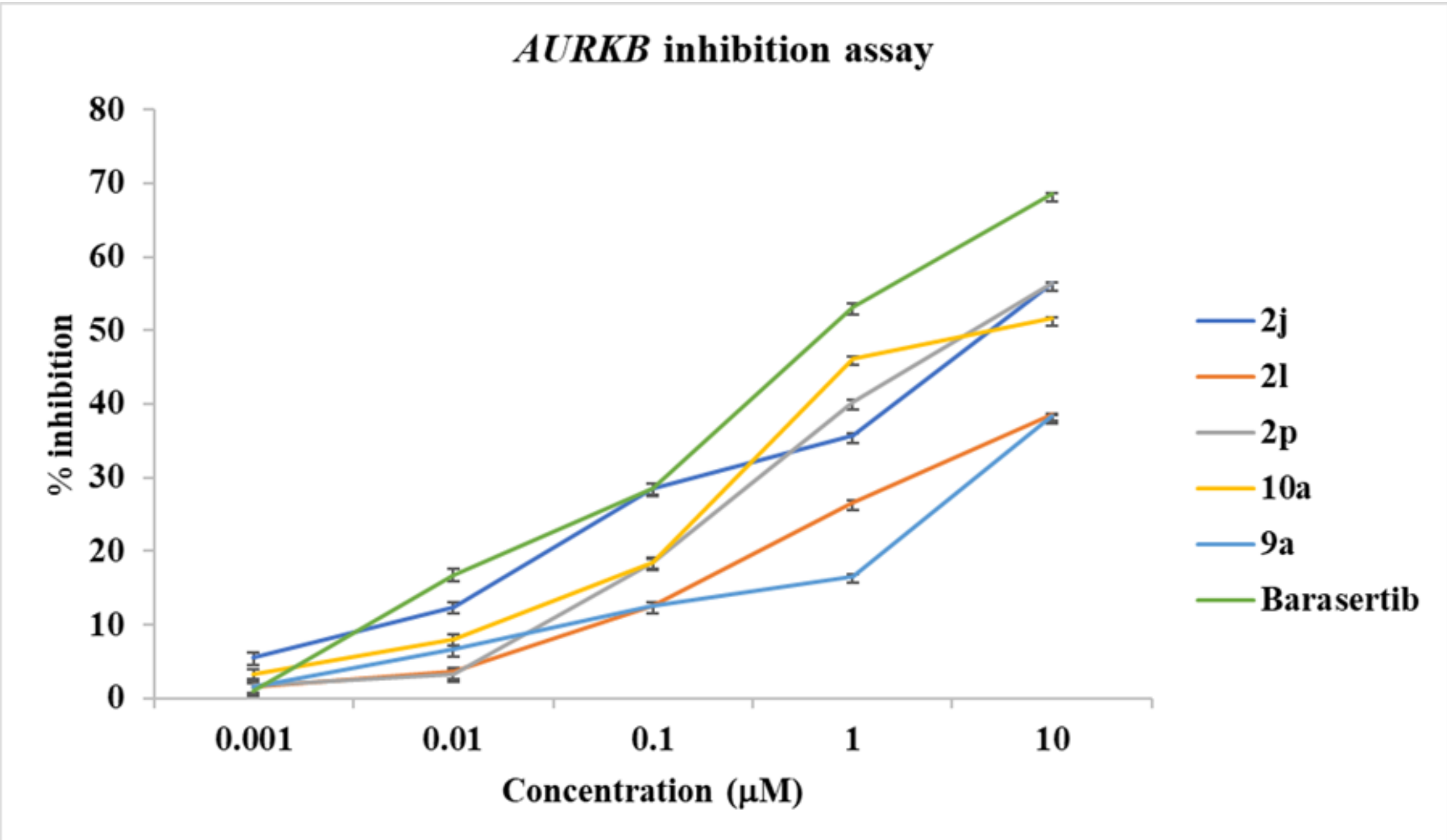

Supplement: Supplementary file 1 [file EXCLI-25-1316-s-001.pdf]
